# Versatile DNA Hydrogel‐Mediated Delivery of Ginsenoside‐Encapsulated Small Extracellular Vesicles to Boost Diabetic Wound Repair

**DOI:** 10.1002/advs.202522920

**Published:** 2026-01-15

**Authors:** Jianming Xing, Shuangyang Li, Yuning Wang, Xushuang Jia, Ruiting Lin, Xintong Liu, Dongxu Wang, Ning Cui, Peng Ji, Jiaqi Chen, Shengnian Wang, Guangzhe Li, Ye Teng, Da Liu, Ye Jin

**Affiliations:** ^1^ Changchun University of Chinese Medicine Changchun China; ^2^ Northeast Asia Research Institute of Traditional Chinese Medicine Changchun University of Chinese Medicine Changchun China; ^3^ Public Experimental Center Changchun University of Chinese Medicine Changchun China; ^4^ Shanghai Key Lab of Reproduction and Development Shanghai Key Lab of Female Reproductive Endocrine Related Diseases Obstetrics & Gynecology Hospital Fudan University Shanghai China; ^5^ Laboratory Animal Center College of Animal Science Jilin University Changchun China; ^6^ Institute For Micromanufacturing Louisiana Tech University Ruston LA USA

**Keywords:** diabetic wound healing, DNA hydrogel, extracellular vesicle, ginseng saponin, mesenchymal stem cell

## Abstract

Diabetic wound healing is often hindered by poor outcomes, prolonged recovery, and high recurrence. To address this, a new therapy approach was demonstrated in this study, in which ginsenoside (GS) molecules are incorporated into small extracellular vesicles (sEV) secreted by mesenchymal stem cells (MSCs), and the formed complexes are then anchored in DNA hydrogels via aptamer‐CD63 affinity as “GS/sEV@DNAgels”. Besides the tissue‐restorative ability that sEVs inherit from MSCs, in GS/sEV@DNAgels, GS molecules provide a superior antimicrobial/anti‐inflammatory environment at wound sites, while DNA hydrogels serve as wound dressings to ensure sustained release kinetics and enhanced skin penetration. An innovative ultrasonic stimulation was developed to promote the massive production of sEVs. By triggering multiple cellular responses that alter membrane fluidity, calcium levels, and relevant protein expression, our approach achieves a 57.7‐fold increase in sEV yield. The synergistic effects of GS and sEVs enhance cell viability, migration, and angiogenesis, as well as local anti‐inflammatory and antibacterial conditions during diabetic wound healing. The upregulation of miR‐424/322 is confirmed as an essential mechanism of this GS/sEV@DNAgel system in accelerating skin restoration. Our work provides a new and promising strategy for diabetic tissue regeneration.

## Graphical abstract

This study presents a DNA hydrogel‐mediated delivery system, in which ginsenoside (GS) molecules are incorporated into small extracellular vesicles (sEV) secreted by mesenchymal stem cells (MSCs) and the formed complexes are then anchored in DNA hydrogels via aptamer‐CD63 affinity as “GS/sEV@DNAgels”, to improve diabetic wound repair. An innovative ultrasonic stimulation approach enabled the massive production of sEVs needed for diabetic skin repair, and the GS/sEV@DNAgels system promotes angiogenesis, suppresses inflammation, and inhibits infection through sustained release and miR‐424 upregulation, offering a synergistic strategy for diabetic tissue regeneration.

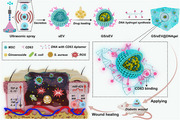

## 1 Introduction

As the primary defense shield, skin protects the human body from pathogenic invasion. But its fragile nature often causes various forms of damage. Unlike minor skin damage that can heal via self‐regeneration, complicated injuries, such as diabetic wounds, delay tissue repair [1]. The hyperglycemic microenvironment in diabetes mellitus triggers the generation of reactive oxygen species (ROS), detrimental inflammation and/or infection, and impairs cell proliferation and critical angiogenesis [2, 3, 4]. These challenges easily lead to slow wound healing, high susceptibility to further complications, and high recurrence and amputation rates [5]. With a rapid increase in the number of diabetic patients, this has emerged as a prevalent global health issue, causing substantial economic and social burdens to patients [6, 7, 8].

Traditional plants or herbs such as ginseng (with bioactive component, ginsenosides or GS) have demonstrated remarkable therapeutic benefits for wound healing, including anti‐inflammatory and antibacterial properties, as well as promotion of tissue regeneration and ROS reduction [9, 10, 11]. However, clinical translation of ginsenosides faces substantial challenges due to their poor solubility, low bioavailability, and inability to efficiently penetrate biological barriers, which significantly compromise therapeutic efficacy [12]. Therefore, loading ginsenosides into some drug delivery vehicles, such as small EVs (sEVs) or exosomes, could provide excellent protection and effective delivery of these poorly soluble drugs [13, 14].

The sEVs are a group of membrane‐enclosed structures of 50–200 nm that are formed by the inward budding of endosomal membranes inside cells. As endogenous substances, sEVs have several advantages as drug delivery vehicles, including efficient crossing of biological barriers and cellular entry, superior compatibility with the immune system, and lower toxicity [15, 16]. Besides their drug carrier advances, sEVs also carry various nucleic acids and proteins from host cells that facilitate signal transmission and regulate multiple biogenesis and physiological conditions of recipient cells [17]. For example, sEVs secreted by mesenchymal stem cells (MSCs) inherit the regenerative potential of the host cells, with therapeutic benefits of alleviating inflammation, stimulating angiogenesis, and enhancing tissue regeneration [18, 19]. Despite these advantages, sEVs secreted through normal metabolism are, however, quite limited in numbers (∼10^7^ mL^−1^), which restricts their promising potential of clinical use in many therapeutic applications [20]. The small size and superior penetration of sEVs also limit their direct use in wound repair due to their short residence time and rapid clearance from the treated sites [19].

Acellular dermal matrices (ADMs) or wound dressings are therefore necessary for diabetic wound healing to host therapeutic drugs and the delivery vehicles. This helps create a sustained release and prolonged therapeutic microenvironment near the wound sites to maximize the drug effectiveness in the promotion of cell proliferation and control of inflammation. Hydrogels are widely used as ADMs/wound dressings because of their excellent moisture retention, biocompatibility, and customizable physical structure [21, 22]. They also serve as sustained‐release platforms to achieve prolonged therapeutic outcomes at wound sites to minimize the need for frequent administration in clinical settings [23]. Among various hydrogels, DNA hydrogels not only preserve all desired hydrogel properties but also offer additional advantages, such as precise tuning of macroporous structures, numerous and specific molecular recognition sites (targeting), and shape adaptation through convenient DNA coding [24, 25, 26].

**FIGURE 1:**
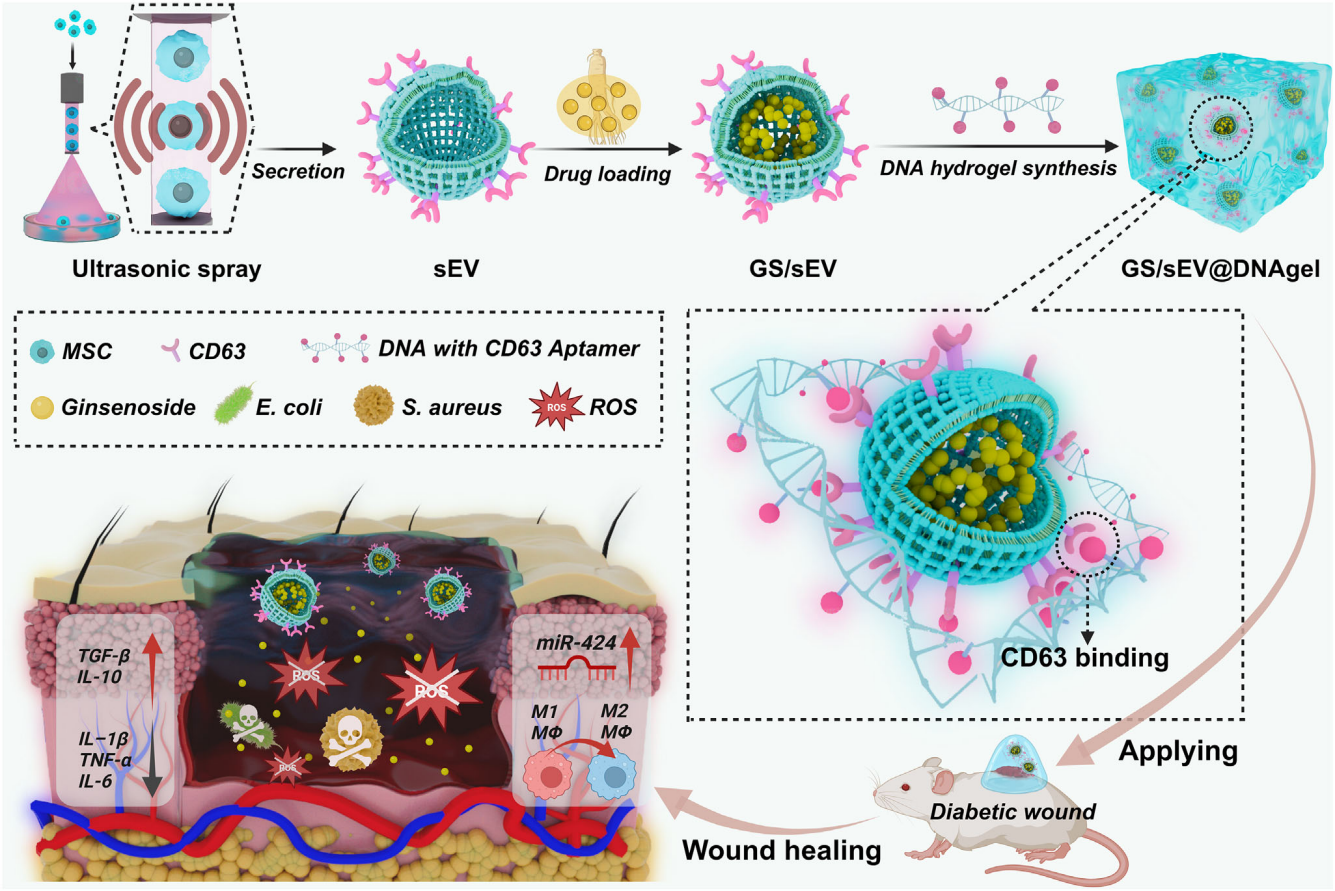
Schematic illustration of GS/sEV@DNAgel generation and its promotion on diabetic wound healing. A novel ultrasonic stimulation method was first employed for the massive production of mesenchymal stem cell‐derived small extracellular vesicles (sEV) while well preserving cell viability. Ginsenosides (GS) were then loaded into those sEVs through an optimized freeze‐thaw protocol to form GS/sEV complexes. The GS/sEV complexes were then incorporated into DNA hydrogels through CD63‐aptamer affinity. As for diabetic wound healing, the GS/sEV@DNAgel system offers the following benefits: (i) inhibits bacterial proliferation, (ii) suppresses inflammatory reactions, and (iii) upregulates miR‐424 to promote angiogenesis.

Inspired by these findings, we prepared sEVs derived from MSCs with ginsenosides encapsulated to create a versatile delivery and therapy complex (Figure 1). By further integrating these complexes into DNA hydrogels, we investigated their potential to promote diabetic wound healing. Specifically, an innovative ultrasonic stimulation approach was first employed to produce sEVs from MSCs with uniform treatment and high yield. Ginsenosides (GS) were then efficiently encapsulated into those sEVs as drug delivery complexes (designated as “GS/sEV”). A CD63 aptamer‐functionalized DNA hydrogel was further synthesized, in which the aptamers capture GS/sEVs via CD63 surface markers to form a wound dressing and a sustained drug‐release system (designated as “GS/sEV@DNAgel”). The therapeutic effects of GS/sEV@DNAgel were then investigated both in vitro and in vivo, with the mechanism validated in a gene‐knockout mouse model. Our work not only establishes a new therapeutic strategy for diabetic wound healing but also provides valuable insights into various regenerative therapies.

## 2 Results

### 2.1 Massive Production of sEVs by Ultrasonic Stimulation and GS Loading in sEVs

**FIGURE 2:**
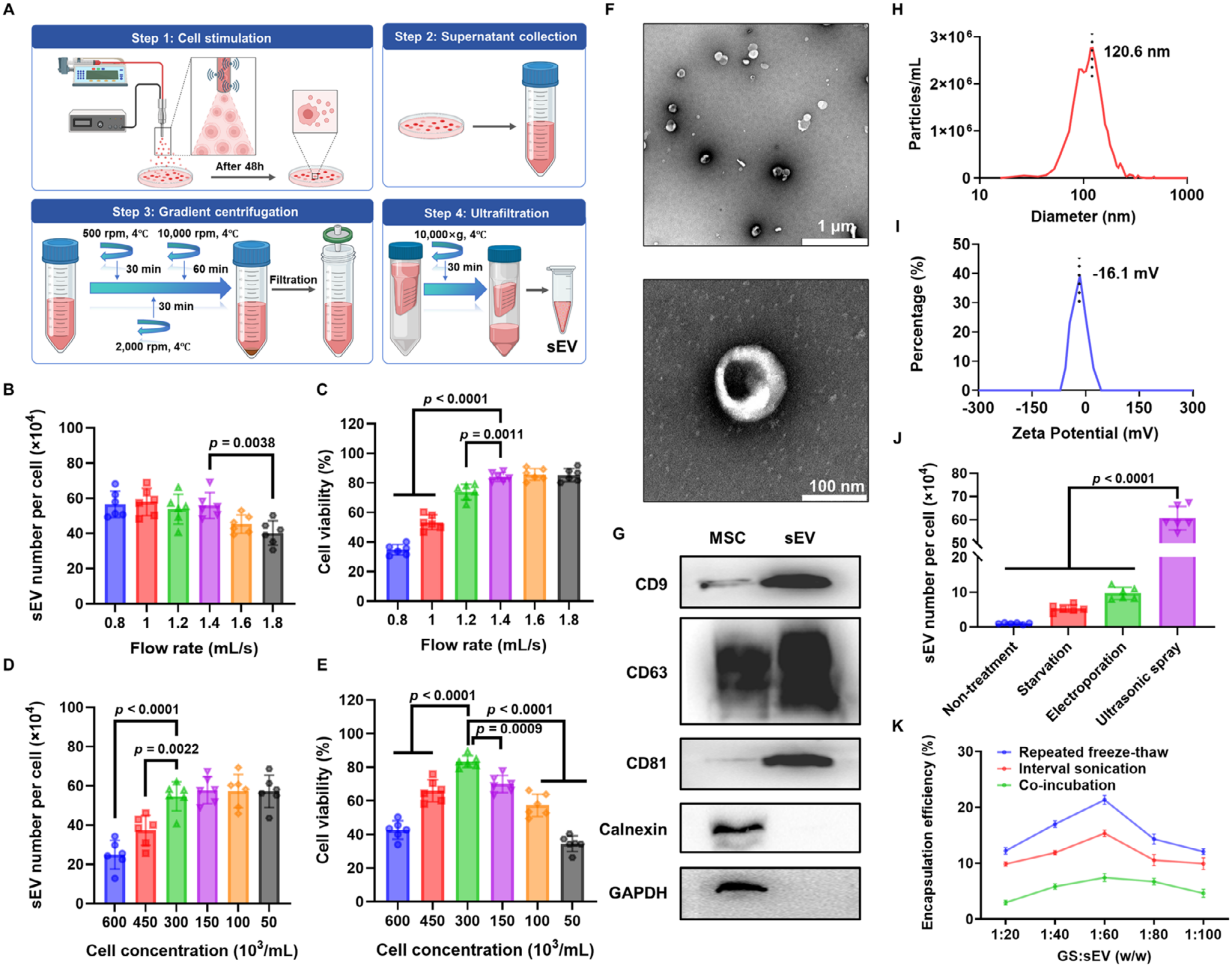
Massive production of sEVs by ultrasonic stimulation and GS Loading. (A) Schematic illustration of sEV stimulation and isolation. (B,C) sEV yield and viability of MSCs treated by ultrasonic stimulation in various flow rates (0.8 to 1.8 mL s^−1^, with an optimal flow rate of 1.4 mL s^−1^; *n* = 6) and (D,E) cell concentration (50 000 to 600 000 cells mL^−1^, with an optimal 300 000 cells mL^−1^; *n* = 6) under a compromise of yield and viability. (F) Transmission electron microscope (TEM) images showing a typical sEV morphology. (G) Western blot characterization confirming the presence of sEV markers (CD9, CD63, and CD81) and absence of endoplasmic reticulum marker Calnexin. (H) NTA measurement of sEVs reveals an average particle size of 120.6 nm. (I) Dynamic Light Scattering (DLS) analysis shows a ζ‐potential of ‐16.1 mV for those sEVs. (J) Comparative sEV yields across different methods detected by NTA (*n* = 6). Ultrasonic stimulation facilitated an 11.35‐fold increase in sEV production relative to starvation stimulation and a 6.28‐fold increase relative to electroporation. (K) Ginsenoside (GS) encapsulation efficiency through different loading methods (*n* = 3). Repeated freeze‐thaw demonstrated the best performance. Data were presented as mean ± SD, and statistical significance was calculated by one‐way ANOVA with Tukey's multiple comparisons test.

To efficiently generate MSC‐derived sEVs, a novel ultrasonic stimulation method was developed (Figure 2A). Specifically, MSCs were resuspended and rapidly passed through the hollow tube of an ultrasonic sprayer to experience stimulation. After further culture of those stimulated MSCs for 48 h, the supernatant of the culture medium was collected and subjected to gradient centrifugation, filtration, and ultrafiltration to isolate secreted sEVs. Key ultrasonic stimulation parameters (frequency, cycles, flow rate, and cell concentration) were optimized to maximize the sEV yield and cell viability with the following conditions eventually adopted: an ultrasonic frequency of 30 kHz (Figure S1), one treatment cycle (Figure S1), a flow rate of 1.4 mL s^−1^ (Figure 2B,C), and a cell concentration of 300 000 cells mL^−1^ (Figures 2D,E). The Nanoparticle Tracking Analysis (NTA) measurements showed significant enhancement of the sEV production by our method, with a yield increase of 11.35‐fold when compared to the starvation stimulation and 6.28‐fold over electroporation, respectively (Figure 2J). A classical cup‐shaped bilayer membrane structure with clear, rounded edges was revealed by transmission electron microscope (Figure 2F). These sEVs have an average size of 120.6 nm (Figure 2H) and a ζ‐potential of −16.1 mV (Figure 2I). Western blot analysis further validated that these sEVs are enriched on markers such as CD63, CD9, and CD81, while being negative for calnexin, an endoplasmic reticulum‐resident protein not normally found in sEVs (Figure 2G). Following the massive sEV production, we also introduced ginsenoside (GS) into those sEVs to form multifunctional GS/sEV complexes with various loading methods. As shown in the results of liquid chromatography‐mass spectrometry (LC‐MS) (Figure S2), the encapsulation efficiency under the repeated freeze‐thaw method (with a GS: sEV ratio varying from 1:20–1:100, w/w) was found significantly higher than that of spaced sonication or co‐incubation methods (Figure 2K). Despite possible loss of sEVs, the freeze‐thaw method (with a GS:sEV ratio of 1:60, w/w) achieved 1.39‐ and 2.87‐ fold increases in GS encapsulation compared with interval sonication (15.4%) and co‐incubation (7.4%), respectively. This innovative ultrasonic stimulation method indeed enables large‐scale production of sEVs from MSCs. GS was also effectively loaded into secreted sEVs to form GS/sEV complexes with multiple desirable properties for diabetic wound healing.

### 2.2 GS/sEV Promotes Cell Migration and Angiogenesis In Vitro

**FIGURE 3:**
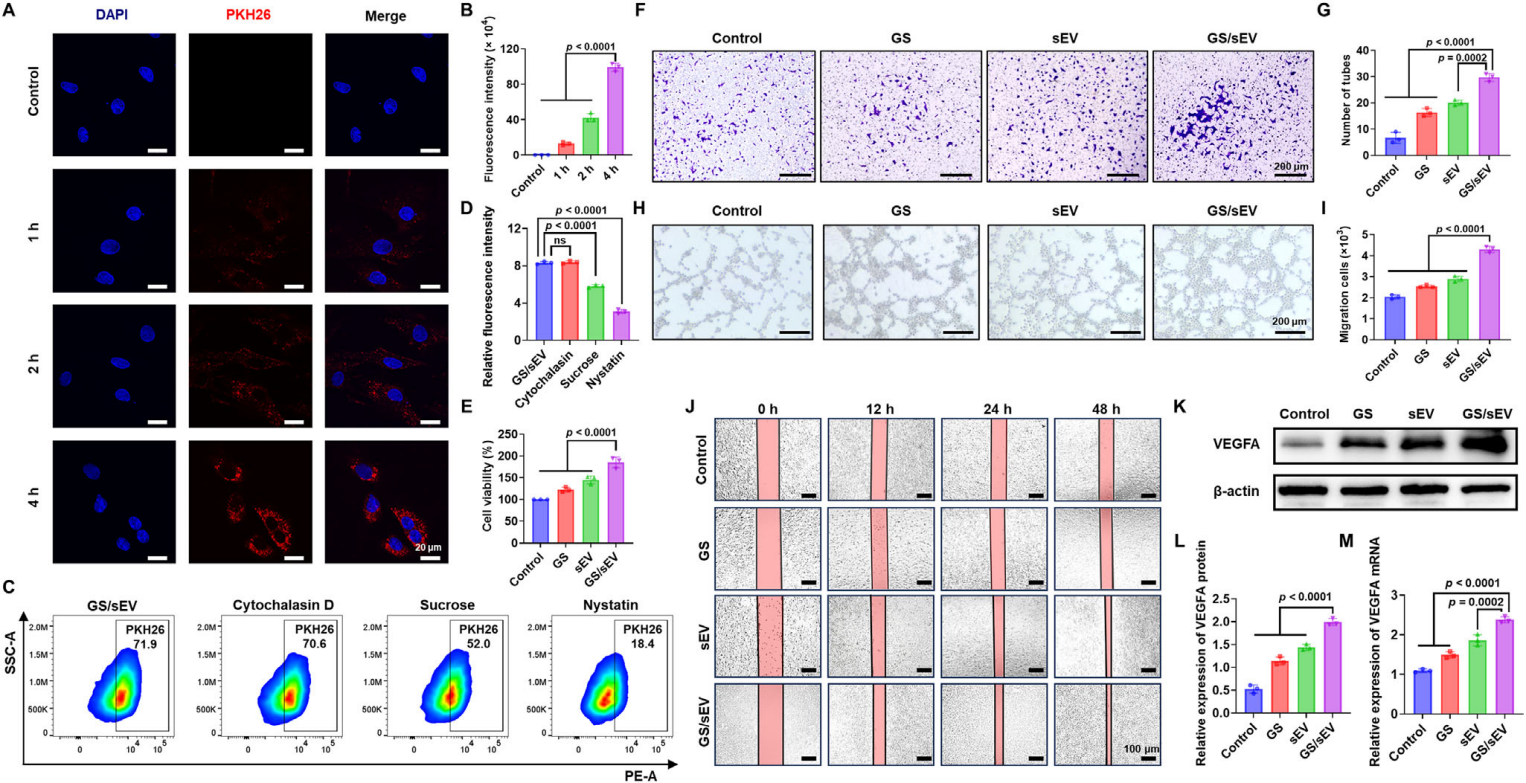
GS/sEV in vitro Promotion on cell migration and angiogenesis. (A,B) Cellular uptake of GS/sEV complexes by HaCaT cells within 4 h, detected by laser scanning confocal microscope (red: PKH26‐labeled GS/sEV; blue: DAPI nuclei staining; scale bar: 20 µm). (C,D) Flow cytometry analysis of GS/sEV internalization mechanism, revealing that lipid raft‐dependent endocytosis is the predominant uptake pathway. (E) Viability of the treated HaCaT cells assessed by CCK‐8 assay. (F,G) Transwell assays on HaCaT cell migration at 12, 24, and 48 h (representative images and quantified migration rates; scale bar: 200 µm). (H,I) Tube formation assay of HUVECs on Matrigel (representative images and branch quantification; scale bar: 200 µm). (J) Wound healing assay for HaCaT cells observed under microscope at 12, 24, and 48 h (scale bar: 200 µm). (K,L) VEGFA protein expression in HUVECs was measured by Western blot assay, band intensities were quantified using ImageJ software, and the expression of VEGFA was normalized to β‐actin. (M) The relative VEGFA mRNA levels in HUVECs measured by RT‐qPCR (normalized to GAPDH; *n* = 3). Data were presented as mean ± SD, and statistical significance was calculated by one‐way ANOVA with Tukey's multiple comparisons test.

To investigate the cellular uptake of GS/sEVs, PKH26‐labeled GS/sEV complexes were first incubated with HaCaT cells. The confocal microscopy results confirm that the GS/sEVs were successfully internalized by cells within 4 h (Figure 3A,B). To reveal the uptake mechanism, multiple inhibitors were used to block different uptake pathways (Cytochalasin D‐Macropinocytosis; Sucrose‐Clathrin; Nystatin‐Lipid rafts) before the addition of PKH26‐labeled GS/sEV complexes. By quantifying the proportion of positive cells using flow cytometry, nystatin was found to significantly reduce the uptake of GS/sEVs (Figure 3C,D), suggesting that lipid‐raft‐mediated endocytosis is the primary mechanism of their internalization. To mimic the diabetic skin microenvironment in vitro, a high‐glucose condition was established. The GS‐to‐sEV mass ratio was first optimized using a CCK‐8 assay, with an optimal ratio of 1:60 (Figure S3). Furthermore, GS/sEV treatment increases HaCaT cell viability by 85.1% (Figure 3E) and HUVEC proliferation by 66.2% (Figure S4) compared to the control (untreated). Since cell migration is crucial for wound healing, transwell assays were conducted further to evaluate the migratory abilities of HaCaT and HUVEC cells. All groups treated with GS, sEVs, and GS/sEVs exhibit promotion of cell migration, with the GS/sEV treatment most effective (enhancing HaCaT migration by 2.60‐fold, as shown in Figure 3F,G, and HUVEC migration by 2.33‐fold, as shown in Figure S5). The scratch assay further confirms such migratory enhancement, with nearly complete closure (99.20%) in HUVECs (Figure S7) and 81.52% in HaCaT cells (Figure 3J, Figure S6) after 48 h treatment with GS/sEV complexes, which vastly outperforms the cases treated with GS or sEV alone. Given the critical roles of angiogenesis in wound repair, we also assessed vascular tube formation of the treated cells on Matrigel. Again, HUVECs treated by the GS/sEV complexes had significantly more tubes than the other groups (4.45‐fold increase when compared to the control groups), indicating superior pro‐angiogenic activity of the GS/sEV complexes (Figure 3H,I). Given that vascular endothelial growth factor A (VEGFA) is a key protein involved in angiogenesis, the expression of VEGFA was also evaluated after drug administration. The GS/sEV treatment indeed boosted VEGFA expression, yielding a 1.99‐fold increase in VEGFA protein levels (Figure 3K,L) and a 2.38‐fold increase in the corresponding mRNA levels (Figure 3M), consistent with results from other pro‐angiogenic assays. In summary, the GS/sEV complexes show efficient cell internalization, which promotes cell migration, angiogenesis, and in vitro diabetic wound healing.

### 2.3 GS/sEV Inhibits Apoptosis and Inflammatory Responses In Vitro

**FIGURE 4:**
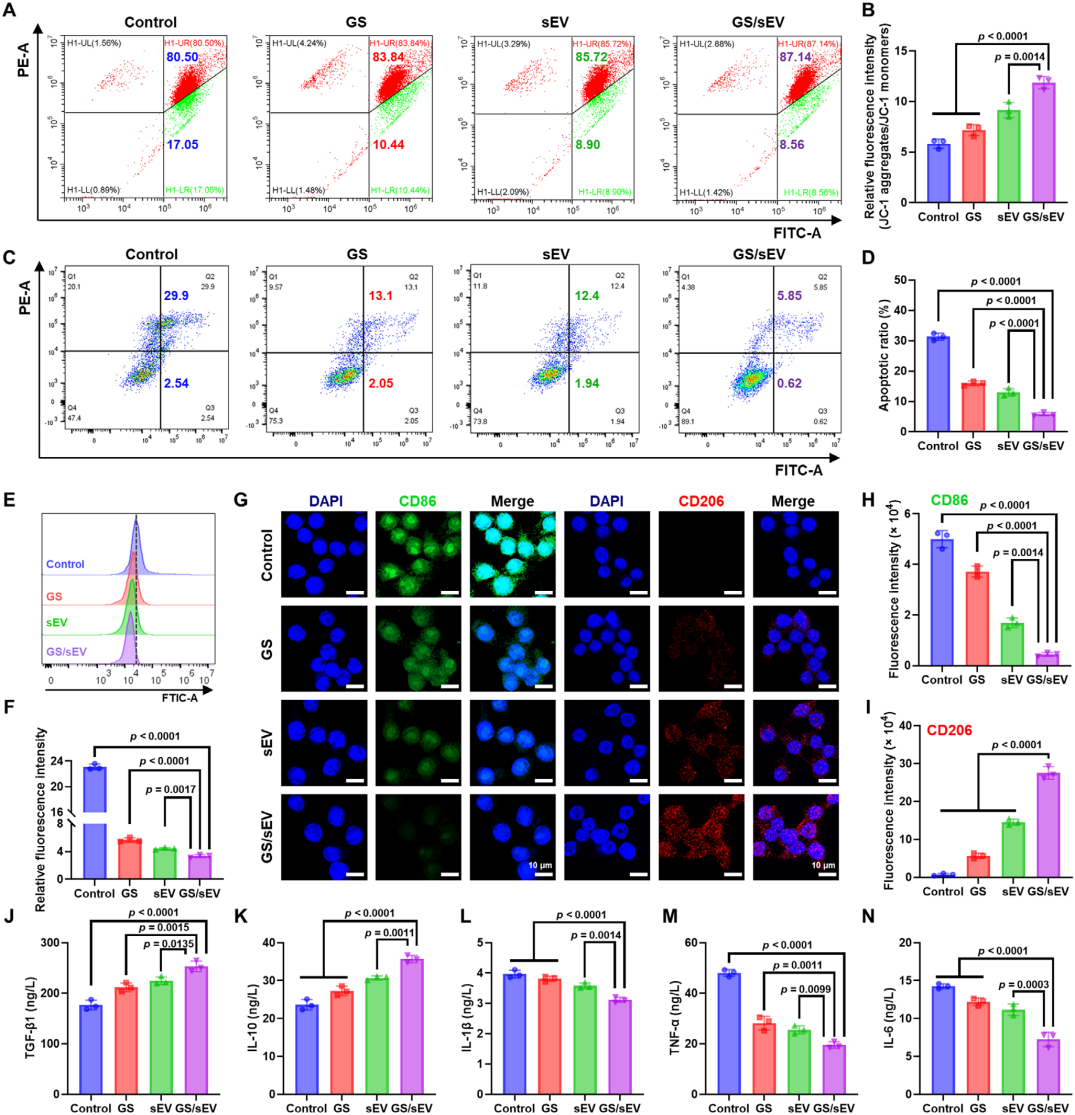
GS/sEV in vitro inhibitions on apoptosis and inflammatory responses. (A,B) Measurement of mitochondrial membrane potential changes (JC‐1 aggregates/JC‐1 monomers) in HaCaT cells analyzed by flow cytometry (A: dot plots; B: relative fluorescence intensity of each group, *n* = 3). (C,D) Apoptosis rates of HaCaT cells in different treatment groups were analyzed by flow cytometry (*n* = 3). (E) Changes in ROS levels in HaCaT cells analyzed by flow cytometry. (F) Relative fluorescence intensity of ROS (FITC) of each group (*n* = 3). (G) The level of CD86 and CD206 in RAW264.7 cells determined by laser scanning confocal microscope (green: CD86; red: CD206; blue: DAPI nuclei staining; scale bar: 10 µm) and (H,I) fluorescence intensity analysis of CD86 and CD206 fluorescence intensity in RAW264.7 cells (*n* = 3). (J–N) Levels of cytokines TGF‐β1, IL‐10, IL ‐ 1β, TNF‐α, and IL‐6 in HaCaT cells were evaluated by ELISA (*n* = 3). Data were presented as mean ± SD, and statistical significance was calculated by one‐way ANOVA with Tukey's multiple comparisons test.

With confirmation of GS/sEV promotion on wound repair in vitro, the potential delay of diabetic wound healing by excessive inflammation was further investigated. Since mitochondrial membrane potential (ΔΨm) is critical for cellular homeostasis and closely linked to apoptosis, we first assessed ΔΨm using the JC‐1 probe, as depolarized ΔΨm during apoptosis shifts JC‐1 from red aggregates to green monomers. Under established high‐glucose conditions, the GS/sEV treatment was found to remarkably reduce JC‐1 monomer formation in HaCaT cells (Figure 4A,B) and HUVECs (Figure S8). Flow cytometry analysis of apoptosis confirms that GS/sEV complexes decrease the apoptotic population by 25.45% in HaCaT cells (Figure 4C,D) and by 24.58% in HUVECs (Figure S9), suggesting better cell protection by GS/sEV treatment under high‐glucose conditions. The level of reactive oxygen species (ROS) under the GS/sEV treatment was further evaluated, as excessive ROS accumulation exacerbates inflammation in diabetic wounds. Our results show significant attenuation of intracellular ROS levels in HaCaT cells (Figure 4E,F) and HUVECs (Figure S10) after the introduction of GS/sEVs, supporting its anti‐inflammatory advantages. To evaluate the effect of the GS/sEV treatment on macrophage differentiation, immunofluorescence of pro‐inflammatory M1 markers (CD86) and anti‐inflammatory M2 markers (CD206) were measured. The weak fluorescence signal of the former (CD86, green) and the strong signal of the latter (CD206, red) were clearly observed after the GS/sEV treatment (Figure 4G–I), indicating their effectiveness in suppressing M1 macrophages and inducing differentiation toward M2 anti‐inflammatory macrophages. ELISA analysis of inflammatory cytokines in HaCaT cells corroborates these findings: GS/sEV significantly upregulate the anti‐inflammatory factors (TGF‐β1, IL‐10) while suppressing the pro‐inflammatory factors (IL ‐ 1β, TNF‐α, IL‐6), as shown in Figure 4J–N). These results demonstrate that GS/sEVs exhibit effective anti‐apoptotic, antioxidant, and immunomodulatory properties under high‐glucose conditions, all of which are beneficial for diabetic wound healing.

### 2.4 Development and Characterization of GS/sEV@DNAgel

**FIGURE 5:**
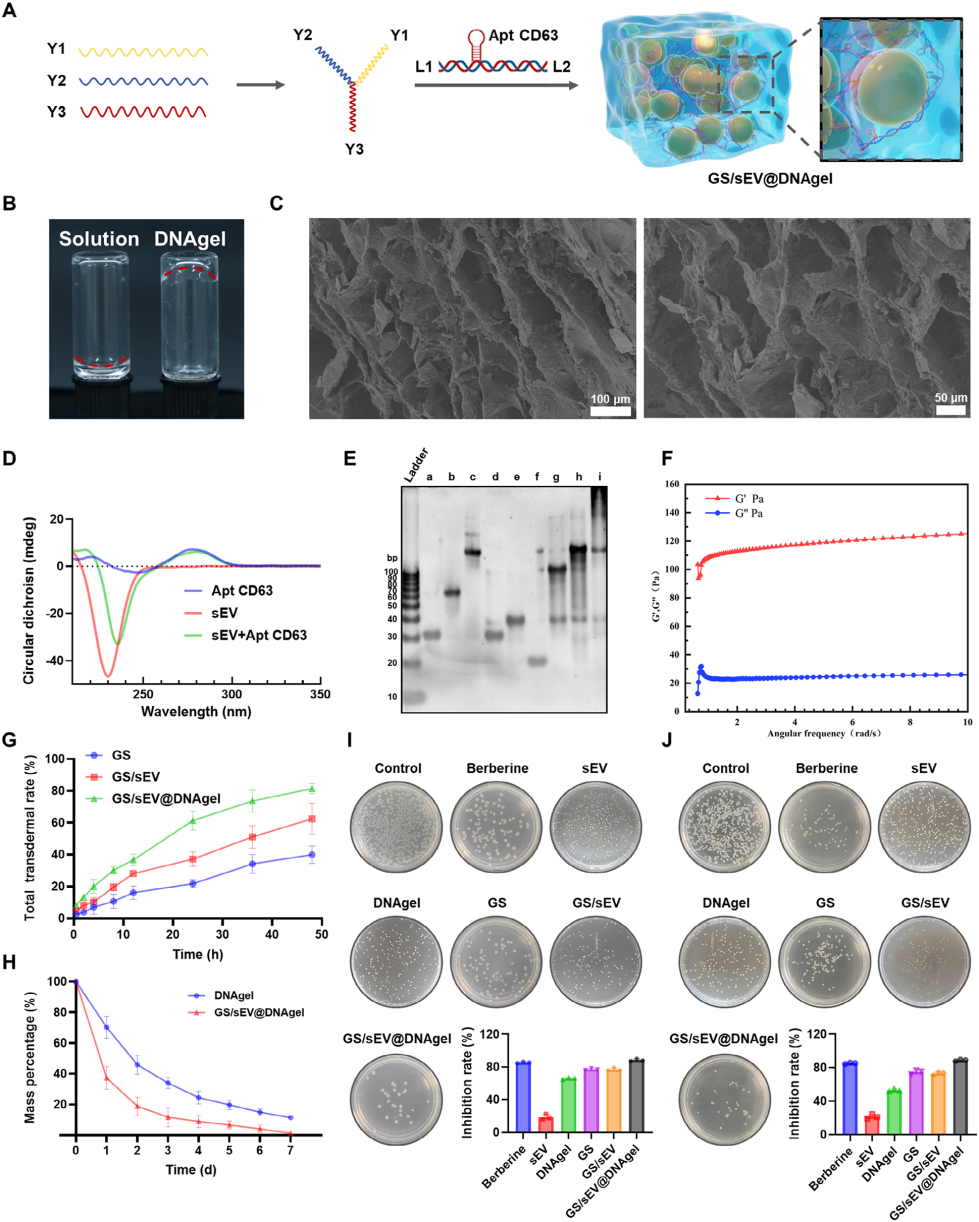
Preparation and characterization of GS/sEV@DNAgels. (A) Schematic illustration of the GS/sEV@DNAgel synthesis process. (B) Photo of the DNA hydrogel states. (C) The macroporous structure of the hydrogel was revealed by scanning electron microscope (scale bars: 100 and 50 µm). (D) The binding interactions between aptamers and sEVs were evaluated by circular dichroism spectroscopy. (E) Agarose gel electrophoresis analysis of the DNA hydrogel formation (lanes: a‐Y1; b‐Y1+Y2; c‐Y1+Y2+Y3; d‐L1‐L2; e‐CD63 aptamer‐L1; f‐L2; g‐L1‐L2+CD63 aptamer‐L1; h‐L1‐L2+CD63 aptamer‐L1+L2; i‐annealed product of Y1+Y2+Y3 and L1‐L2+CD63 aptamer‐L1+L2). (F) Rheological characterization of the DNA hydrogel. (G) Evaluation of drug permeation for free GS, GS/sEV, and GS/sEV@DNAgel over 48 h (*n* = 3). (H) Degradation curves of GS/sEV@DNAgel versus blank hydrogel as measured by weight loss (*n* = 3). (I,J) Representative images and quantitative analysis from the evaluation of GS/sEV@DNAgel's inhibitory effects against *E. coli* and *S. aureus* (*n* = 3). Data were presented as mean ± SD, and statistical significance was calculated by one‐way ANOVA with Tukey's multiple comparisons test.

To further evaluate the in vivo outcomes of the GS/sEV complexes in diabetic wound healing, we first synthesized CD63 aptamer‐conjugated DNA hydrogels. Y1, Y2, and Y3 strands of DNA were mixed at desired ratios to form a cross‐linked hydrogel scaffold, with the CD63 aptamers integrated via L1 and L2 linkers. Since CD63 is highly expressed on the surface of sEVs, GS/sEV complexes were captured by the DNA hydrogels via CD63 aptamers to form an integrated wound dressing and a sustained release system, GS/sEV@DNAgel (Figure 5A). The resulting hydrogels were found to be stable and strong enough under gravity (Figure 5B), and to carry a relatively uniform macroporous microstructure (Figure 5C), which is essential for supporting cell proliferation and facilitating drug transport. Circular dichroism spectroscopy revealed a significant shift in the absorption peaks for the hydrogel‐encapsulated sEVs when compared to sEVs alone, confirming the successful capture of sEVs by DNA hydrogels (Figure 5D). Agarose gel electrophoresis indicated that the annealed GS/sEV complexes migrated much more slowly than the linear templates, suggesting that the DNA sequences had been successfully cross‐linked into hydrogels (Figure 5E). Rheological measurements exhibited typical characteristics of flexible hydrogels, with the storage modulus (G′) predominating over the loss modulus (G″), as shown in Figure 5F. GS/sEV@DNAgels maintain well their structural integrity and stability at ‐20°C throughout the entire four‐week storage testing period (Figure S11).

In the ex vivo percutaneous penetration evaluation, GS/sEV@DNAgel groups showed a higher penetration rate than that of free GS and GS/sEV groups, suggesting that the skin penetration of GS was improved by GS/sEV@DNAgel (Figure 5G, Figures S12 and S13). The in vitro release studies also found that GS/sEV@DNAgel released 86.17% of GS within 48 h, slower than free GS and GS/sEV samples (Figure S14). The GS/sEV@DNAgel also degraded faster than the unloaded hydrogels, which were nearly completed within 7 days (Figure 5H). And the hemolysis rates of the degradation products from both gels remained below 5%, demonstrating their safety in future clinical use (Figure S15). The antibacterial performance of these GS/sEVs/DNgels was further evaluated against the predominant pathogens *E. coli* and *S. aureus*. GS/sEV@DNAgel exhibited 88.49% growth suppression against *E. coli* and 88.93% against *S. aureus* (Figure 5I,J). This confirms the dual roles of these GS/sEV@DNAgels as both drug carriers and antimicrobial agents. Our findings indeed demonstrate that GS/sEV@DNAgels exhibit favorable structural stability, controlled drug release, enhanced skin permeability, and broad antimicrobial activity, all of which are desirable for diabetic wound healing.

### 2.5 GS/sEV@DNAgel In Vivo Promotion on Diabetic Wound Healing

**FIGURE 6:**
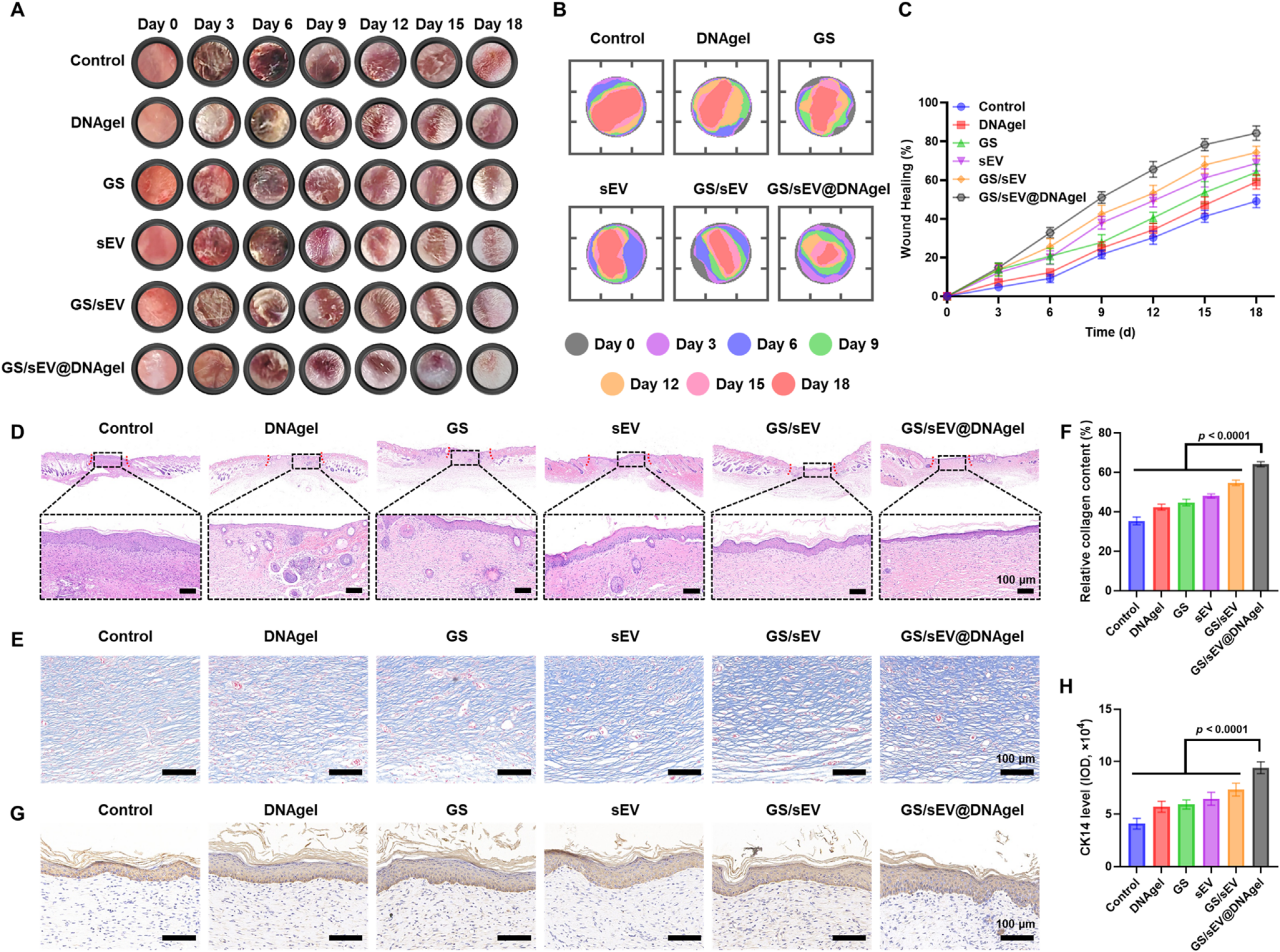
GS/sEV@DNAgel promotion on wound healing in vivo. (A) Representative images showing the progression of wound healing at different stages. (B) Traces of wound closure in different treatment groups. GS/sEV@DNAgel demonstrated the most pronounced closure pattern. (C) Quantitative assessment of final wound closure rates (*n* = 6), revealing GS/sEV@DNAgel achieved 84.23% healing efficiency versus 49.14% in the control group on Day 18. (D) H&E staining of tissue sections in different treatment groups on Day 18 (scale bar: 100 µm). (E) Masson‐stained wound tissue sections as observed under a light microscope (scale bar: 100 µm). (F) Statistical analysis of the collagen fibers’ percentage relative to the total tissue area. (G) Immunohistochemical staining for basal keratinocyte marker CK14 protein in skin tissue. (H) Quantitative analysis of CK14 expression levels in skin tissue samples from each group. Data were presented as mean ± SD, and statistical significance was calculated by one‐way ANOVA with Tukey's multiple comparisons test.

A mouse model of streptozotocin‐induced diabetic wound was established to assess the therapeutic potential of the GS/sEV@DNAgel. The wound was treated every three days for a total period of 18 days, and the healing progress was photographed before each treatment (Figure 6A). Wound area was measured to quantify the wound closure progress in all treatment groups. At the experimental endpoint, the GS/sEV@DNAgel‐treated groups achieved 84.23% closure of the wound area, a 1.71‐fold improvement over the control groups (Figure 6B,C), confirming their superior wound‐healing performance. Histopathological examination via H&E staining provided further evidence of the therapeutic effectiveness of GS/sEV@DNAgels. Although all treatment groups showed some improvement in tissue architecture, the GS/sEV@DNAgel groups showed the most significant improvement, with alleviation of skin structural abnormalities, acanthosis, and inflammatory cell infiltration (Figure 6D). The promotion of GS/sEV@DNAgel to collagen deposition, a critical determinant of wound strength and remodeling, was further evaluated using Masson's staining. Microscopic images revealed that the GS/sEV@DNAgel groups had the densest collagen fiber distribution (Figure 6E), a 1.81‐fold increase in collagen when compared to the untreated groups (Figure 6F). The differentiation and migration of keratinocytes in the basal layer, which lead to the formation of new epidermis, were also found to be accelerated after the GS/sEV@DNAgel treatment. As demonstrated by CK14 immunohistochemical staining, the most extensive CK14 distribution was observed in the GS/sEV@DNAgel groups. In contrast, it was relatively widespread in the GS/sEV groups and partially expressed in the control groups (Figure 6G,H).

### 2.6 GS/sEV@DNAgel Promotion on Angiogenesis and Suppression of Inflammation In Vivo

**FIGURE 7:**
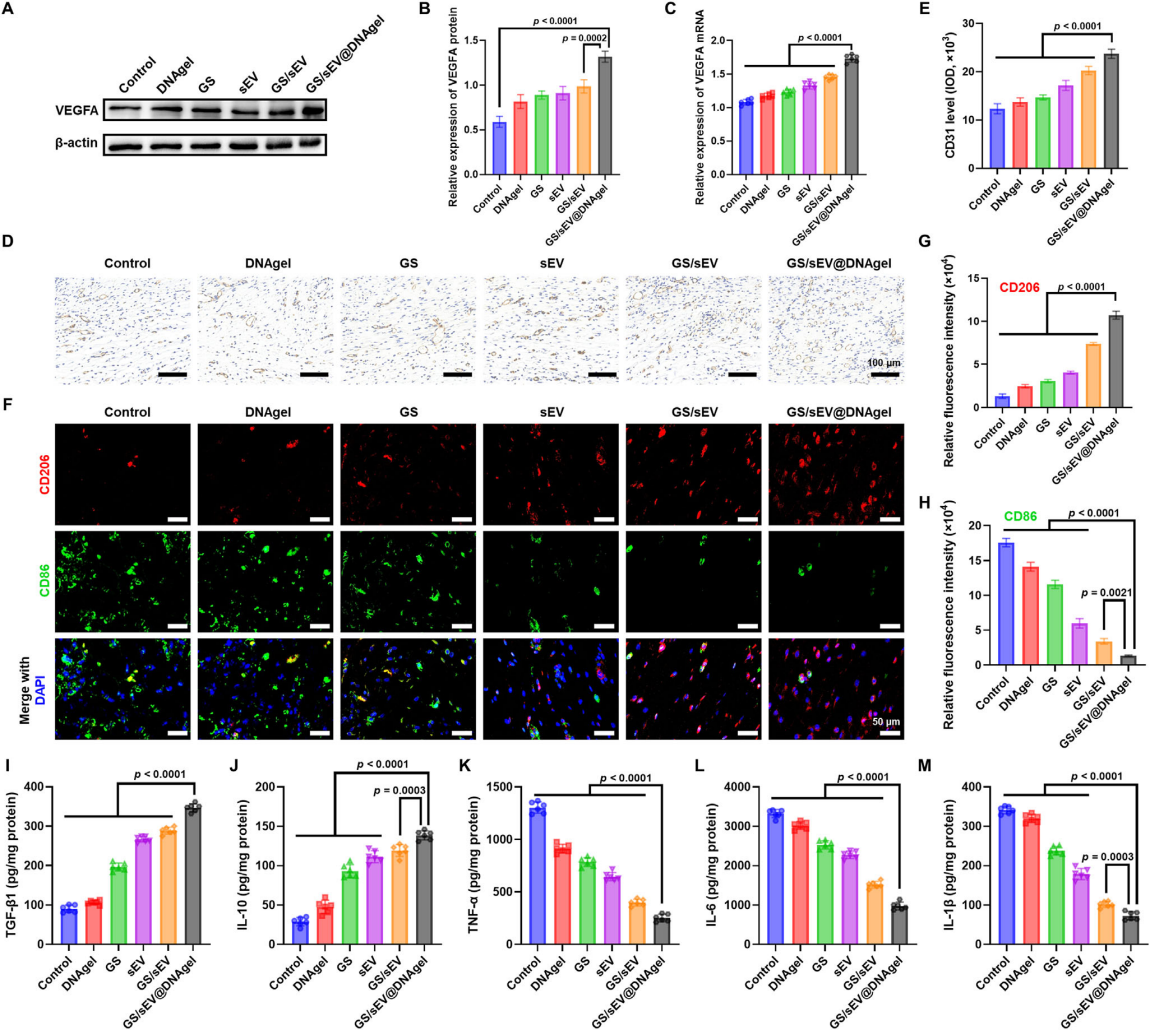
GS/sEV@DNAgel promotes angiogenesis and suppresses inflammation in vivo. (A,B) Western blot of the VEGFA protein expression in skin tissues demonstrated significantly enhanced angiogenic signaling in the GS/sEV@DNAgel groups, with band intensities normalized to β‐actin. (C) The relative VEGFA mRNA levels in skin tissue detected by RT‐qPCR (normalized to GAPDH; *n* = 6), revealing a 1.60‐fold upregulation of the mRNA levels in the GS/sEV@DNAgel group. (D) CD31 expression in skin tissue from each group was assessed by immunohistochemistry. (E) Quantitative analysis of CD31 expression levels. (F) The levels of CD86 and CD206 in skin tissue determined by dual immunofluorescence staining (green: CD86; red: CD206; blue: DAPI nuclei staining; scale bar: 50 µm). (G,H) Analysis of CD86 and CD206 fluorescence intensity. (I–M) Levels of cytokines in skin tissues evaluated by ELISA (*n* = 6), confirming the therapeutic immunomodulation of the GS/sEV@DNAgel through the TGF‐β1/IL‐10 elevation and TNF‐α/IL‐6/IL‐1β suppression. Data were presented as mean ± SD, and statistical significance was calculated by one‐way ANOVA with Tukey's multiple comparisons test.

Angiogenesis plays a pivotal role in wound healing by supplying nutrients for the regenerated tissues. Vascular endothelial growth factor A (VEGFA), a key regulator of angiogenesis, was therefore quantified in our studies. Western blot analysis showed that the GS/sEV@DNAgel treatment increased VEGFA protein expression by 1.62‐fold compared with the untreated control groups (Figure 7A,B). At the same time, the RT‐qPCR results further confirmed a similar upregulation of VEGFA mRNA levels (Figure 7C), corroborating the robust angiogenic stimulation by the GS/sEV@DNAgels. These findings were further validated by assessing newly formed blood vessels using CD31 immunohistochemistry (Figure 7D). Compared with the control groups, CD31 expression increases 1.93‐fold in the GS/sEV@DNAgel groups (Figure 7E), confirming their promotion of angiogenesis. Given the critical roles of macrophage polarization in diabetic wound resolution, we also evaluated the immunomodulatory effects of GS/sEV@DNAgel in vivo. Immunofluorescence co‐staining of CD206 (M2 anti ‐ inflammatory macrophage marker, red) and CD86 (M1 pro‐inflammatory macrophage marker, green) revealed that GS/sEV@DNAgel indeed elevated the M2 polarization by 8.20‐fold and suppressed the M1 macrophages by 13.06‐fold (Figure 7F–H). The cytokine levels in skin tissue also corroborate these observations: for the GS/sEV@DNAgel groups, the anti‐inflammatory factors (TGF‐β1, IL‐10) were significantly upregulated, while the pro‐inflammatory factors (TNF‐α, IL‐6, IL‐1β) were largely suppressed (Figure 7I,M). The levels of these cytokines in serum showed no significant changes, suggesting low immunogenicity of GS/sEV@DNAgel (Figure S16). The unchanged liver and kidney function indicators (AST, ALT, Cr, and BUN) after administration further confirm its low systemic toxicity (Figure S17). With these in vivo studies in a diabetic wound mouse model, we concluded that GS/sEV@DNAgels can accelerate collagen production and angiogenesis, enhance keratinocyte differentiation, and suppress inflammation. The superior pro‐angiogenic, pro‐regenerative, and immunomodulatory properties of the GS/sEV@DNAgels make them attractive for clinical use in diabetic wound repair.

### 2.7 MiR‐424 Mediates the Therapeutic Effects of GS/sEV@DNAgels in Diabetic Wound Healing

**FIGURE 8:**
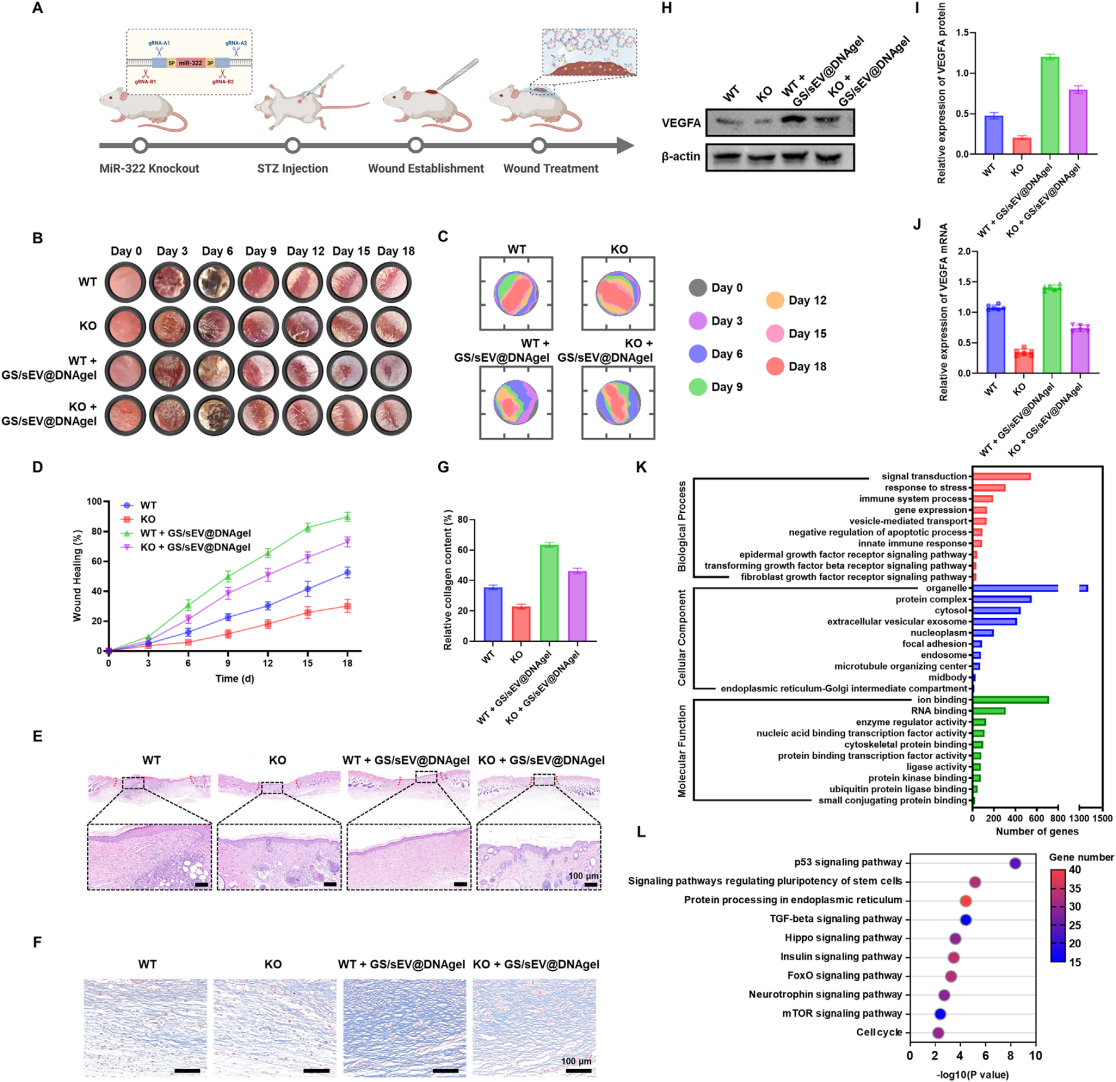
MiR‐424 mediates the therapeutic performance of GS/sEV@DNAgels in diabetic wound healing. (A) Schematic illustration of miR‐322 knockout (KO) diabetic mice generation. (B) Representative photographs documenting the wound healing progression at different stages. (C) Traces of wound area reduction in different treatment groups. (D) Assessment of final wound closure rates (*n* = 6). (E) H&E staining of tissue sections in different treatment groups on Day 18 (scale bar: 100 µm). (F) Masson's staining of wound tissue, revealing collagen distribution (scale bar: 100 µm). (G) Percentage of collagen‐positive area relative to the total tissue area. (H,I) Western blot assay and quantitative analysis of VEGFA protein expression in skin tissues, band intensities were quantified using ImageJ software, and the expression of VEGFA was normalized to β‐actin. (J) The relative VEGFA mRNA levels in skin tissues detected by RT‐qPCR (normalized to GAPDH; *n* = 6). (K,L) Gene Ontology (GO) annotations and Kyoto Encyclopedia of Genes and Genomes (KEGG) pathway enrichment highlighting the involvement of miR‐424 in wound repair. Data were presented as mean ± SD, and statistical significance was calculated by one‐way ANOVA with Tukey's multiple comparisons test.

MiR‐424 has been found to regulate cell proliferation and migration [27, 28]. To reveal the possible changes of miR‐424 during the GS/sEV treatment, we measured miR‐424 levels in high‐glucose‐treated HaCaT cells via RT‐qPCR. The GS/sEV treatment indeed increases miR‐424 levels in HaCaT cells by 1.92‐fold relative to untreated controls (Figure S18). Subsequently, the inhibition of miR‐424‐5p significantly suppressed cell migration in the scratch assay, while the addition of GS/sEVs partially alleviated such inhibitory effects in vitro (Figure S19). To further confirm these findings in vivo, a diabetic wound mouse model with miR‐322 (the human miR‐424 homolog) knockout was established (Figure 8A). Knockout of miR‐322 (KO) also significantly inhibited wound healing in diabetic mice, with the wound healing rates in the KO group slowed down by 33.95% compared to the wild‐type (WT) mice (Figure S20). To confirm that this delay is related to impaired angiogenesis, VEGFA expression in the WT and KO mice was assessed via Western blot and RT‐qPCR. Knockdown of miR‐322 resulted in a 64.9% reduction in the VEGFA protein levels (Figure S21) and a 48.84% decrease in the VEGFA mRNA levels (Figure S22) in skin tissue. Given that miR‐322/424 significantly suppresses wound healing in diabetes, we further evaluated whether it is a target of GS/sEV@DNAgels in diabetic wound treatment. Diabetic mice were grouped as follows: WT, KO, WT + GS/sEV@DNAgel, and KO + GS/sEV@DNAgel. First, wound healing at 18 days was recorded: WT + GS/sEV@DNAgel group exhibited the highest healing rate (89.81%), while the KO group had the lowest (30.14%). However, the therapeutic effect of GS/sEV@DNAgels was suppressed in KO mice, with a 16.73% reduction in healing rate in KO + GS/sEV@DNAgel mice compared to WT + GS/sEV@DNAgel mice (Figure 8B–D). H&E staining of skin tissue post‐treatment revealed that following miR‐322 knockout, the skin exhibited more severe structural abnormalities than the WT group, manifested as incomplete keratinization and thickening of the spinous layer. GS/sEV@DNAgels partially mitigated the disruption of skin architecture (Figure 8E). The blue color in Masson's staining indicated collagen content in the skin. Compared to WT + GS/sEV@DNAgel, the collagen area in KO + GS/sEV@DNAgel decreased by 27.10% (Figure 8F,G). Similarly, CK14 immunohistochemical staining results showed that miR‐322 knockdown suppressed the differentiation of basal‐layer keratinocytes (Figure S23). In further assessments, miR‐322 knockdown inhibited the angiogenic effects of GS/sEV@DNAgel, as evidenced by the following changes in the KO + GS/sEV@DNAgel group compared to the WT + GS/sEV@DNAgel group: VEGFA protein expression decreased by 33.33% (Figure 8H,I), mRNA levels dropped by 47.27% (Figure 8J), and CD31 expression decreased by 31.41% (Figure S24). Finally, we analyzed the function of miR‐424 through Gene Ontology (GO) annotations and Kyoto Encyclopedia of Genes and Genomes (KEGG) pathway analyses (Figure 8K,L). It was found that miR‐424 participates in biological processes such as immune regulation, vesicle transport, and the inhibition of apoptosis. MiR‐424 regulates multiple signaling pathways, including those mediated by the epidermal growth factor receptor, transforming growth factor beta receptor, and fibroblast growth factor receptor. These pathways are all closely related to wound healing. Therefore, upregulation of miR‐424 is one primary mechanism in our GS/sEV@DNAgel treatment that accelerates diabetic wound healing, while the promotion arises from multiple pathways.

## 3 Discussion

In this study, we developed an innovative GS/sEV@DNAgel system to promote diabetic wound healing. Specifically, our approach addresses the limitation on sEV quantity using a new ultrasonic stimulation platform. Unlike previous studies by Zheng et al. [29], in which an ultrasonic transducer was directly pointed toward the bottom of cell culture dishes, yielding a 40‐fold increase in sEV release, our innovative approach has MSCs flow through the hollow tube of an ultrasonic sprayer, enabling massive production of sEVs without sacrificing cell viability. This increases the yield of sEVs by 57.7‐fold compared with natural secretion, 11.35‐fold with starvation stimulation, and 6.28‐fold with electroporation stimulation, respectively. Besides the high yield, our method also allows uniform stimulation of the host cells by flowing them through the same stimulation field to minimize the batch‐to‐batch variability during sEV production. To reveal the mechanisms underlying this innovative stimulation approach for sEV release, we examined several cellular processes, including membrane fluidity, calcium channels, autophagy, and changes in relevant protein expression (e.g., P62 and Rab GTPases) before and after the ultrasonic treatment. A significant rise in the cellular calcium ion level was found after the stimulation (Figure S25), which has been reported to promote the accumulation of the Endosomal Sorting Complexes Required for Transport (ESCRT) complex, thereby facilitating sEV secretion [30]. Following the stimulation, MSCs also exhibit increased cell membrane fluidity (Figure S26), which allows easier membrane budding, ESCRT diffusion and assembly, and endocytosis that promote the release and uptake of sEV [31]. Western blot analysis further revealed the downregulated P62 level in MSCs (Figure S27), thereby enhancing autophagy, which is closely associated with exosome secretion [32]. We also observed an upregulation of Rab27b (Figure S27), a key regulator that promotes the docking of multivesicular endosomes and sEV secretion when overexpressed [33]. In brief, this new ultrasonic stimulation triggers multiple cellular processes that promote the sEV secretion.

In addition to the tissue‐regenerative ability inherited from MSCs, the MSC‐derived sEVs also carry GS to offer additional therapeutic benefits. GS was loaded into sEVs via repeated freeze‐thaw cycles to form multifunctional GS/sEV complexes. The in vitro uptake studies confirmed the efficient internalization of these GS/sEV complexes by cells via lipid raft‐mediated endocytosis. Upon uptake of GS/sEVs, recipient cells exhibit a significant reduction of ROS levels and enhancement of cell viability, migration, and angiogenesis. GS/sEV complexes can also reprogram macrophages from a pro‐inflammatory M1 phenotype toward an anti‐inflammatory M2 phenotype, accompanied by the downregulation of TNF‐α, IL‐6, and IL‐1β and upregulation of TGF‐β1 and IL‐10. Despite the advances of GS in reducing ROS and acquiring anti‐inflammatory and antibacterial properties, our current loading method for GS in sEVs is not perfect. Repeated freeze‐thaw cycles could cause damage to sEVs. Specifically, a 20% decrease in protein and a 31% decrease in RNA were observed in sEVs after loading (Figure S28A), consistent with earlier studies reporting reduced exosome numbers and degradation of internal nucleic acids [34]. HaCaT viability assays also revealed a 5.84% reduction in cell survival upon treatment with freeze‐thaw sEV samples compared to untreated controls (Figure S28B). However, the freeze‐thaw approach provides higher GS dosage than other available loading methods, and the GS/sEVs prepared in this way outperformed co‐administered free GS + sEVs during in vitro and in vivo therapeutic studies. Alternative loading strategies that do not damage sEVs might be needed in the future to further promote this GS/sEV@DNAgel system in diabetic wound healing.

To apply in vivo wound healing, we further engineered DNA hydrogels with aptamers on the surface to allow the GS/sEV encapsulation via aptamer‐CD63 affinity. DNA hydrogels offer advantages such as biological safety, well‐defined microporous structures, and low immunogenicity [35]. Unlike some chemical modification methods, in which aptamers were immobilized on DNA chains via click chemistry [36], the annealing hybridization approach allows aptamers to be grafted to DNA hydrogels without biocompatibility difficulty for in vivo therapeutic applications [37]. In this work, we constructed a CD63 aptamer‐modified DNA hydrogel to selectively recruit sEVs through the affinitive CD63‐aptamer interactions. The formed GS/sEV@DNAgels serve as both a wound dressing and a controlled release system. The in vitro release and transdermal studies demonstrated sustained drug release kinetics and enhanced skin penetration of the GS/sEV@DNAgels compared with free GS or the GS/sEV groups. Superior promotion of angiogenesis, as well as antimicrobial and anti‐inflammatory benefits, was also observed, with their synergistic acceleration of diabetic wound healing.

The RT‐qPCR analysis revealed elevated miR‐424 levels in GS/sEV‐treated HaCaT cells. Inhibition of miR‐424 impaired HUVEC migration under high‐glucose conditions, whereas GS/sEVs partially restored this deficit. MiR‐424 has been reported to be associated with angiogenesis. Teng et al. demonstrated its role in hepatocellular carcinoma, where the MYLK‐AS1/miR‐424‐5p/E2F7 axis modulated vascular endothelial growth factor receptor 2 (VEGFR2)‐dependent vascularization [38]. The GS/sEV@DNAgel system is suggested to accelerate diabetic wound repair through miR‐424/322‐mediated angiogenesis and M2 macrophage polarization to suppress inflammation. To better understand the therapeutic mechanism of GS/sEV with miR ‐ 424, miR‐322 (the mouse homolog of human miR‐424) knockout diabetic wound model (KO) was established. KO mice exhibited significantly delayed wound healing, with a 33.95% reduction in wound closure when compared to wild‐type mice. However, the GS/sEV@DNAgel treatment only partially mitigated the healing impairment caused by miR‐322 knockout, consistent with previous in vitro findings of miR‐424 inhibition. These findings suggest that the upregulation of miR‐424/322 is an important mechanism underlying the acceleration of GS/sEV@DNAgels in diabetic wound healing. But other factors must exist. GS has been demonstrated to accelerate angiogenesis through signaling pathways such as PI3K and β‐catenin activation [39, 40], while MSC‐derived sEV primarily acts via AKT and ERK signaling pathways [41]. Additionally, MSC‐derived sEV can suppress high‐glucose‐induced apoptosis in diabetic nephropathy [28]. To further explore potential participation in diabetic nephropathy and retinopathy, GS/sEV complexes were administered to high‐glucose‐treated kidney proximal tubular cells (HK‐2) and retinal Müller cells. The levels of miR‐322 in both cell types were found to be higher after the GS/sEV treatments, as was the cell viability (Figure S29). Given that upregulation of miR‐322 helps alleviate diabetic nephropathy [42] and retinopathy [43], GS/sEV complexes have the potential to treat these conditions. In other words, this innovative approach might benefit other cross‐disease therapies beyond diabetic wound healing.

## 4 Conclusion

In summary, we successfully demonstrate an innovative ultrasonic stimulation approach to promote sEV production from MSCs, yielding 57.7‐fold more than natural secretion, 11.35‐fold more than starvation stimulation, and 6.28‐fold more than electroporation stimulation. After encapsulating GS in sEVs and further integrating the resulting complexes into DNA hydrogels, the new GS/sEV@DNAgel system exhibits a sustained drug release kinetics, enhanced skin penetration, significant reduction in ROS levels, and superior promotion of angiogenesis, antimicrobial, and anti‐inflammatory, all beneficial for their collective acceleration in diabetic wound healing. The leverage of miR‐322/424 levels is an important mechanism underlying the acceleration of GS/sEV@DNAgels in diabetic wound healing, which may also benefit other complicated, cross‐disease applications, such as alleviating diabetic nephropathy and retinopathy.

## 5 Experimental Section

### 5.1 Materials

Mesenchymal stem cells (MSCs), human umbilical cord endothelial cells (HUVECs), human immortalized epidermal cells (HaCaT), mouse macrophage cells (RAW264.7), human kidney proximal tubular cells (HK‐2), and human retinal müller cells were obtained from Procell Biotechnology Co., Ltd. (Wuhan, China). MSCs were cultured in medium MEM‐α (HyClone, USA) supplemented with 10% exosome‐depleted fetal bovine serum (Thermo Fisher Scientific, USA) and 1% penicillin/streptomycin (Thermo Fisher Scientific, USA). HUVECs, HaCaT, RAW264.7, HK‐2, and Müller cells were cultured in DMEM low‐glucose medium (AC01L022, Life‐iLab Biotech, China) supplemented with 10% fetal bovine serum (AC03L055, Life‐iLab Biotech, China) and 1% penicillin/streptomycin (AC03L332, Life‐iLab Biotech, China). All the cells were cultured in 10 cm dishes in a 5% CO_2_ incubator at 37°C, medium was changed every two days. BCA (Bicinchoninic acid) kit (AP12L025) and cell counting kit‐8 (CCK‐8, AC11L054), and siRNA transfection reagent (AC04L052) were purchased from Life‐iLab Biotech Co., Ltd (Shanghai, China). TGF‐β1 ELISA kit (SYP‐M0416), IL‐10 ELISA kit (SYP‐M0033), IL‐1β ELISA kit (SYP‐M0026), TNF‐α ELISA kit (SYP‐M0036), were all purchased from UpingBio Technology Co, Ltd. IL‐6 ELISA kit (EMC004.48) was obtained from Neobioscience Technology Co, Ltd. Aspartate aminotransferase (AST/GOT) activity assay kit (E‐BC‐K236‐M), Alanine aminotransferase (ALT/GPT) activity assay kit (E‐BC‐K235‐M), Creatinine (Cr) colorimetric assay kit (E‐BC‐K188‐M), and urea (BUN) colorimetric assay kit (E‐BC‐K183‐M) were purchased from Elabscience Biotechnology Co., Ltd. (Wuhan, China). Fluo‐4 AM Calcium ion fluorescent probe (S1060) was obtained from Beyotime Biotechnology (Shanghai, China). Cell membrane fluidity assay kit (G&V10362.1) was purchased from JIwei Biological Technology Co., Ltd. (Shanghai, China). Anti‐CD9 (ab92726), anti‐CD63 (ab134045), anti‐CD81 (ab155760), anti‐P62 (ab91526), and anti‐Rab27b (ab76779) antibodies were purchased from Abcam (USA), anti‐Calnexin (AF5362) antibody was purchased from Affinity Biosciences (China), anti‐VEGFA antibody (K001655P) was purchased from Solarbio Science & Technology Co., Ltd. (Beijing, China), and anti‐β‐actin antibody (LF201) was purchased from Epizyme Biotech (Shanghai, China). Reverse Transcription Kit (AG 11711) and SYBR Green qPCR kit (AG11701) were purchased from ACCURATE BIOTECHNOLOGY (HUNAN) Co., Ltd. ICR mice were purchased from Liaoning Changsheng Biotechnology Company (Benxi, China).

### 5.2 Production of sEVs

For ultrasonic stimulation, MSCs were digested, centrifuged at 1000× *g* for 10 min, and re‐suspended in serum‐free OPTI‐MEM medium at a density of 3 × 10^5^ cells mL^−1^. The sample was then passed through an ultrasonic sprayer (JY‐W50, Hangzhou Ultrasonic Equipment, frequency: 30 kHz) at a flow rate of 1.4 L h^−1^. The stimulated cells were then transferred and cultured in exosome‐free medium for 48 h. The cell supernatant was collected and centrifuged at 500 rpm for 30 min, then at 2000 rpm for 30 min to remove cell debris and apoptotic bodies, and finally at 10 000 rpm for 60 min to remove large microvesicles. Subsequently, vesicles larger than 220 nm were removed by passing the sample through a 0.22 µm filter. Finally, sEVs were concentrated using 100 kDa MWCO ultrafiltration by centrifugation at 4000× *g* for 30 min (Figure 2A).

### 5.3 Characterization of sEV

The morphology of sEVs was examined using a transmission electron microscope (TEM) at 100 kV. The particle size and concentration of sEV were quantified using nanoparticle tracking analysis (NTA). The ζ‐potential of sEV was measured by a dynamic light scattering instrument (DLS). Marker proteins were confirmed by Western blot analysis to assess levels of sEV markers (CD9, CD63, CD81) and intracellular proteins (calnexin).

### 5.4 GS Loading in sEVs

GS loading was performed by three different methods: interval sonication, repeated freeze‐thaw, and co‐incubation. (i) Interval sonication: sEV was mixed with GS and sonicated for 5 min, followed by a 5‐min recovery period. This process was repeated 5 times. (ii) Repeated freeze‐thaw: sEV was mixed with GS in a specific ratio, frozen in liquid nitrogen for 5 min, and then immediately thawed in a 37°C‐water bath for 5 min. This cycle was repeated 5 times. (iii) Co‐incubation: MSC‐sEV solution was mixed with GS under continuous agitation. Samples were then purified using 100 kDa MWCO ultrafiltration to remove unencapsulated GS. The best method was determined based on the GS encapsulation efficiency, quantified by Liquid Chromatography‐Mass Spectrometry (LC‐MS).

### 5.5 Preparation and Characterization of DNA Hydrogel

The Y scaffold was prepared by mixing Y1, Y2, and Y3 in a 1:1:1 molar ratio in 0.2 M PB buffer (pH 7.4, 300 mM NaCl). Similarly, the linker was prepared by mixing L1, L2, CD63‐L1, and L2 in a 1:1:1 molar ratio in the same buffer (Sequences are listed in Table S1). These solutions were incubated at 90°C for 10 min, then slowly cooled to room temperature. The Y scaffold and linker were mixed in a 2:3 volume ratio using vortex mixing, and the resulting mixture gelled within minutes. Agarose gel electrophoresis was used to characterize DNA hybridization. First, DNA in hydrogel was dissolved at a concentration of 3 M in a buffer (containing 20 mM Tris‐HCl (pH 7.4), 5 mM MgCl_2_, and 300 mM NaCl). The sample was then incubated at 90°C for 10 min, followed by annealing to 25°C at a cooling rate of ‐1°C min^−1^. The samples were mixed with a 2× loading buffer in a 1:1 ratio and loaded onto an agarose gel for electrophoresis. The electrophoresis buffer was 1× TBE buffer with 10 mM NaCl. After staining with SYBR Gold (PerkinElmer Life Sciences, USA), images were scanned using the iBright FL1000 imaging system. The properties of the hydrogel were also analyzed using a scanning electron microscope and circular dichroism spectroscope.

### 5.6 In Vitro Release Evaluation

GS solution, GS/sEV, and GS/sEV@DNAgel (all containing 200 mg GS) were encapsulated in 50 kDa dialysis bags. The dialysis bags were then placed in 80 mL of PBS buffer and stirred at 37°C and 200 rpm to promote drug release. At designated sampling moments, 1.0 mL of release solution was collected, and 1.0 mL of fresh PBS buffer was then replenished in the dialysis bag. The GS content in the release solution was determined by LC‐MS to generate the drug release curves.

### 5.7 Ex Vivo Skin Permeation Evaluation

The Franz diffusion setup was used to perform the ex vivo permeation experiment. The system was first filled with saline as the receiving medium. Skin from the back of mice was first collected. After removing the subcutaneous layer, a piece of skin was then mounted onto the diffusion system with the cuticle facing up and the dermis down. GS solution, GS/sEV, and GS/sEV@DNAgel (all containing 200 mg GS) were then placed in the upper chamber on top of the delimited skin. The system was then sealed and maintained in a 37°C water bath for diffusion tests. At 0.5, 2, 4, 8, 12, 24, 36, and 48 h, 1 mL of medium was collected for testing, and the same volume of fresh receiving medium was added. The collected medium was mixed with 0.5 mL of acetonitrile to break the emulsion and centrifuged to collect the supernatant. The supernatant was replenished to 2 mL with methanol and passed through a 0.22 µm filter. The quantity of GS in the filtrate was measured by LC‐MS. The skin permeability, skin retention, and total permeability (skin permeability + skin retention) were calculated.

### 5.8 Stability Evaluation of Hydrogel

Equal amounts of DNA hydrogel and GS/sEV@DNAgels were freeze‐dried, and the initial dry weight was recorded as W1. After soaking the gels in PBS at 37°C for 7 days, the dry weight of each sample (W2) was measured daily. The mass change percentage was then calculated as (W2/W1) × 100%.

### 5.9 Evaluation of Antibacterial Property of Hydrogel

The growth curves of *E. coli* and *G. aureus* in LB broth were generated by measuring the optical density at 600 nm (OD600). During the logarithmic phase, cultures were diluted, and their OD600 was measured. This value was then used to calculate the OD600 corresponding to a concentration of 1 × 10^8^ CFU mL^−1^. Subsequently, the culture solution at 1 × 10^8^ CFU mL^−1^ was diluted 10^5^‐ or 10^6^‐fold and spread onto agar plates under different treatments, followed by incubation at 37°C. Finally, colonies were photographed, counted, and analyzed using ImageJ software.

### 5.10 Cell Viability Assay

HaCaT cells and HUVECs were seeded at 1 × 10^4^ cells per well in 96‐well plates. After 24 h, glucose was added to the medium at a final concentration of 60 mM, and the cells were incubated for an additional 24 h to establish the high‐glucose model. The cells were then incubated with PBS (control group), GS, sEVs, or GS/sEVs for 24 h. Subsequently, 20 µL of CCK‐8 reagent was added to each well, and the absorbance at 450 nm was measured after an additional 30 min of incubation. Cell viability was expressed as the percentage of absorbance relative to unmodeled cells.

### 5.11 Cellular Uptake Assay

HaCaT cells were seeded in 6‐well plates at a density of 8 × 10^5^ cells per well. After 24 h, glucose was added to the medium (final concentration 60 mM), and cells were treated for an additional 24 h to establish the high‐glucose model. PKH26 (Umibio, China) labeled GS/sEV was added and incubated with cells for 1, 2, and 4 h. After incubation, cells were washed twice with PBS and fixed with 4% formaldehyde for 15 min. Nuclei were stained with DAPI (Solarbio, China), and fluorescence was observed and recorded using a Laser Scanning Confocal Microscope (LSCM, Zeiss 980, Germany).

### 5.12 Wound Healing Assay

HaCaT cells and HUVECs were seeded at a density of 5 × 10^5^ cells per well in 6‐well plates. Once 100% confluence was reached, glucose was added to the medium (final concentration, 60 mM), and cells were further maintained for 24 h to establish the high‐glucose model. Then the monolayers in each well were scraped with a 200 µL plastic pipette tip and washed three times with PBS. The cells were treated with PBS (control group), GS, sEVs, or GS/sEVs for 48 h. Images were captured using an inverted microscope (Olympus, Japan) at 0, 12, 24, and 48 h. Wound area was measured using ImageJ software to calculate cell migration rates.

### 5.13 Cell Migration Assay

Cell migration was assessed using an 8‐µm transwell (NEST, USA). A total of 4 × 10^5^ high‐glucose‐treated cells were seeded in serum‐free medium with PBS (control group), GS, sEVs, or GS/sEVs in the upper chamber of a transwell, allowing migration into the lower chamber. After 48 h of incubation, the cells on the lower surface of the membrane were fixed with 4% paraformaldehyde for 30 min, washed, and stained with 0.1% crystal violet for 30 min. The cells were imaged and counted using ImageJ software.

### 5.14 Angiogenesis Assay

Each well of a 24‐well plate was coated with matrix gel (BD Pharmingen) and polymerized at 37°C for 30 min. High‐glucose‐treated HUVECs were harvested after 12 h of starvation and incubated at a density of 30,000 cells per well for 6 h. Images were captured under a microscope. Tube formation was quantified by counting the number of junctions.

### 5.15 Western Blot

Protein samples were lysed with RIPA Lysis Buffer (AR0010, Shanghai Acmec Biochemical Technology), supplemented with 1% protease inhibitor cocktail (NCM Biotech, P001), according to the manufacturer's instructions. The protein concentration was determined using a BCA kit. Protein samples were separated using 10% SDS‐PAGE and transferred onto polyvinylidene fluoride (PVDF) membranes (Millipore). The membranes were blocked for 1 h with 5% non‐fat milk in Tris‐buffered saline (TBS) at room temperature and incubated overnight at 4°C with primary antibodies. The membranes were washed three times and incubated with a secondary antibody conjugated to horseradish peroxidase for 45 min at room temperature. The PVDF membranes were developed using a chemiluminescence detection system, and the images were analyzed using ImageJ software.

### 5.16 Real‐Time Quantitative PCR Assay

VEGFA mRNA expression in HUVECs was measured by real‐time quantitative PCR (RT‐qPCR). Total RNA was isolated from HUVECs using the Cell RNA Extraction Kit (N066, NJJCBIO) and reverse transcribed into cDNA using the Reverse Transcription Kit. Gene expression was quantified using the SYBR Green qPCR kit. The expression levels of VEGFA were normalized to GAPDH. The primer sequences are shown in Table S2.

### 5.17 Measurement of Cellular Reactive Oxygen Species

HaCaT cells and HUVECs were plated in 6‐well plates at a density of 5 × 10^5^ cells per well. When cells reached approximately 70% confluence, they were serum‐starved in serum‐free medium for 12 h. Glucose solution (60 mM) was then added for 24 h to induce modeling. Each well was then treated with PBS (control group), GS, sEVs, or GS/sEVs for 24 h. After treatment, the cells were trypsinized with EDTA‐free trypsin and washed twice with pre‐chilled PBS. Reactive oxygen species (ROS) levels were measured using the ROS assay kit (Beyotime, China) according to the manufacturer's protocol. Briefly, the washed cells were resuspended in 1 mL of DCFH‐DA (10 µmol/L) and incubated at 37°C for 20 min. The cells were then centrifuged at 1,000× *g* for 5 min and washed three times with PBS. Cells were analyzed by flow cytometry (Beckman, USA) using the FITC channel.

### 5.18 Cell Apoptosis Assay

HaCaT cells and HUVECs were plated in 6‐well plates at a density of 5 × 10^5^ cells per well. Glucose solution (60 mM) was then added for 24 h to induce modeling. Each well was then treated with PBS (control group), GS, sEVs, or GS/sEVs for 24 h. After treatment, the cells were trypsinized with EDTA‐free trypsin and washed twice with pre‐chilled PBS. Apoptosis was quantified using the Annexin V ‐ FITC Apoptosis Detection Kit (AC10860, Shanghai Acmec Biochemical Technology Co., Ltd) according to the manufacturer's protocol. Briefly, the washed cells were resuspended in 100 µL of 1× binding buffer and incubated with 5 µL of PI and 5 µL of annexin V‐FITC at room temperature for 15 min. Then, 400 µL of 1× binding buffer was added to each tube, and the samples were analyzed by flow cytometry (Beckman, USA).

### 5.19 Measurement of Mitochondrial Membrane Potential Changes

HaCaT cells and HUVECs were plated in 6‐well plates at a density of 5 × 10^5^ cells per well. Glucose solution (60 mM) was then added for 24 h to induce modeling. Each well was then treated with PBS (control group), GS, sEVs, or GS/sEVs for 24 h. After treatment, the cells were trypsinized with EDTA‐free trypsin and washed twice with pre‐chilled PBS. Mitochondrial membrane potential was assessed using the mitochondrial membrane potential assay kit (Beyotime C2006) according to the manufacturer's instructions. Briefly, the washed cells were resuspended in 0.5 mL of JC‐1 solution and incubated at 37°C for 20 min. After that, the cells were centrifuged at 600 g for 5 min and washed with binding buffer. Finally, the samples were analyzed by flow cytometry (Beckman, USA).

### 5.20 Polarization of M1 Phenotype Macrophages

RAW264.7 cells were seeded at a density of 1 × 10^4^ cells per well in 12‐well plates and cultured at 37°C for 24 h. Following 24‐hour treatment with 200 ng mL^−1^ lipopolysaccharide (LPS) to induce M1 polarization, the medium was replaced with a fresh one. Cells were then incubated with PBS (Control group), GS, sEVs, or GS/sEVs for 48 h. Subsequently, immunofluorescence assays were performed to examine changes in the M1 macrophage marker CD86 and the M2 macrophage marker CD206. Specifically, after drug administration, cells were washed with PBS and fixed with paraformaldehyde. They were then incubated overnight with anti‐CD86 antibody (DF6332, Affinity) and anti‐CD206 antibody (DF4149, Affinity), respectively. After incubation with primary antibodies, cells were then incubated with an Alexa Fluor 488‐conjugated (green) or Alexa Fluor 454‐conjugated (red) secondary antibody for 1 h. Nuclei were stained with DAPI (Solarbio, China), and fluorescence was observed and recorded using a Laser Scanning Confocal Microscope (LSCM, Zeiss 980, Germany).

### 5.21 Inflammatory Factor Assay

HaCaT cells were plated in 6‐cm dishes at a density of 1 × 10^6^ cells per well. A glucose solution (60 mM) was added for 24 h to induce modeling. Each well was then treated with PBS (control group), GS, sEVs, or GS/sEVs for 24 h. The cells were lysed with RIPA Lysis buffer (Beyotime, China) and then centrifuged at 12 000 rpm for 10 min. The supernatant was collected, and the levels of TNF‐α, IL‐1β, IL‐6, IL‐10, and TGF‐β1 were measured using the ELISA kits according to the manufacturer's instructions.

### 5.22 Inhibition of MiR‐424‐5p

HUVECs were seeded at a density of 5 × 10^5^ cells per well in 6‐well plates. After 24 h, glucose was added to the medium (final concentration, 60 mM), and the cells were incubated for an additional 24 h to establish the high‐glucose model. Then, 100 nM miR‐424‐5p inhibitor or negative control inhibitor (MedChemExpress) was mixed with 20 µL Transfection Reagent (MedChemExpress) and incubated with cells for 6 h. Cells were then subjected to a wound healing assay.

### 5.23 Establishment of the Mouse Diabetic Wound Model

Male ICR mice (aged 6 weeks) were purchased from Liaoning Changsheng Biotechnology Co., Ltd. They were fed under standard feeding conditions. All animal experiments were conducted in accordance with the guidelines approved by the Experimental Animal Welfare Ethics Committee of Jilin University (Approval No. SY202503050). To induce diabetes, the mice were acclimatized for 7 days and then fasted for 12 h before intraperitoneal injection of streptozotocin (STZ) at a dose of 150 mg kg^−1^. The mice continued to receive a high‐sugar, high‐fat diet after the injection. On day 7, blood glucose levels were measured at random, and successful diabetes induction was defined as a random blood glucose level of 16.7 mmol L^−1^. The successfully induced diabetic mice were randomly assigned to six groups: PBS (Control), DNAgel, GS, sEV, GS/sEV, and GS/sEV@DNAgel. The backs of the mice were shaved, sterilized, and circular skin excision wounds (6 mm in diameter) were created. To minimize the effects of skin contraction on wound healing, an inner‐diameter rubber ring and skin adhesive were used to secure the wound site. Different treatments were administered to each group, and wound images were captured every three days. Wound area (in mm^2^) was measured using ImageJ software and used to calculate the healing rates. After treatment, wound tissues were collected for immunohistochemistry, H&E staining, and Masson staining. Skin tissues were lysed and collected for Western blot (WB) and RT‐qPCR analysis, as previously described.

### 5.24 Establishment of miR‐322 Knockout Mouse Model

The miR‐322 (the mouse homolog of human miR‐424) knockout mouse model was generated using CRISPR/Cas9‐mediated genome editing. The miR‐322 gene (gene ID: 723907; miRBase: MI0000590) in mice is located on the X chromosome, with the mature miR‐322 site selected as the target for knockout. Two pairs of sgRNAs were designed and cloned into targeting vectors, which were subsequently sequenced for validation. The sequences were listed in Table S3. In vitro‐transcribed Cas9 mRNA and gRNA were coinfected into fertilized eggs to generate knockout (KO) mice. The pups were genotyped by PCR, followed by sequencing analysis to confirm the knockout.

### 5.25 Functional Analysis of miR‐424

The function of miR‐424 was analyzed using the DIANA‐miRPath v3.0 online analysis software (http://www.microrna.gr/miRPathv3) [44]. Species: Human, miRNA ID: hsa‐miR‐424‐5p, p‐value threshold: 0.05, Enrichment Analysis Method: Fisher's Exact Test. Gene Ontology (GO) and Kyoto Encyclopedia of Genes and Genomes (KEGG) pathway enrichment analyses were performed separately, and the results were visualized using GraphPad Prism 10.0 (USA).

### 5.26 Statistical Analysis

Results are presented as mean ± SD. To ensure reproducibility of results, each experiment was repeated at least three times. Differences were analyzed using one‐way ANOVA with Tukey's multiple comparisons test. *p* values less than 0.05 were considered statistically significant, with *p* < 0.01 and *p* < 0.001 indicating highly and extremely significant differences, respectively.

## Author Contributions

All authors were involved in this work. J.X., S.L., and Y.W. were responsible for Conceptualization, Validation, and Writing – Original Draft. X.J., R.L., P.J., and J.C. were responsible for Methodology and Data Curation. X.L., D.W., and N.C. were responsible for Formal analysis. Y.T. was responsible for Visualization. S.W. and G.L. were responsible for Resourses, Investigation, and Writing – Review & Editing. D.L. Y.J. were responsible for Project Administration and Funding Acquisition. All authors approved the final manuscript.

## Conflicts of Interest

The authors declare no conflict of interest.

## Supporting information




**Supporting File**: advs73854‐sup‐0001‐SuppMat.docx.

## Data Availability

The data that support the findings of this study are available from the corresponding author upon reasonable request.
