# Supplementary material for: Versatile DNA Hydrogel‐Mediated Delivery of Ginsenoside‐Encapsulated Small Extracellular Vesicles to Boost Diabetic Wound Repair
Source: Adv Sci (Weinh). 2026 Jan 15;13(25):e22920. doi: 10.1002/advs.202522920 (PMC13137787; doi:10.1002/advs.202522920)
Supplement: Supplementary file 1 — Supporting File: advs73854‐sup‐0001‐SuppMat.docx. [file ADVS-13-e22920-s001.docx]

**Versatile DNA Hydrogel-Mediated Delivery of Ginsenoside-Encapsulated Small Extracellular Vesicles to Boost Diabetic Wound Repair**

Jianming Xing^1, 2, 3#^, Shuangyang Li^1, 2, 3#^, Yuning Wang^4#^, Xushuang Jia^1, 2, 3^, Ruiting Lin^1, 2, 3^, Xintong Liu^1, 2, 3^, Dongxu Wang^5^, Ning Cui^1, 2^, Peng Ji^1, 2, 3^, Jiaqi Chen^1, 2, 3^, Shengnian Wang^6*^, Guangzhe Li^1, 3*^, Ye Teng^1, 3*^, Da Liu^1, 3*^, Ye Jin^1, 2*^

^1^ Changchun University of Chinese Medicine, Changchun 130117, China

^2^ Northeast Asia Research Institute of Traditional Chinese Medicine, Changchun University of Chinese Medicine, Changchun 130117, China

^3^ Public Experimental Center, Changchun University of Chinese Medicine, Changchun 130117, China

^4^ Shanghai Key Lab of Reproduction and Development, Shanghai Key Lab of Female Reproductive Endocrine Related Diseases, Obstetrics & Gynecology Hospital, Fudan University, Shanghai 200433, China

^5^ Laboratory Animal Center, College of Animal Science, Jilin University, Changchun 130062, China

^6^ Institute for Micromanufacturing, Louisiana Tech University, Ruston, LA 71272, USA

#These authors contributed equally

*Corresponding authors: Shengnian Wang: swang@latech.edu; Ye Teng: tengye@ccucm.edu.cn; Guangzhe Li: ligz@ccucm.edu.cn; Da Liu: liuda@ccucm.edu.cn; Ye Jin: jinye@ccucm.edu.cn.


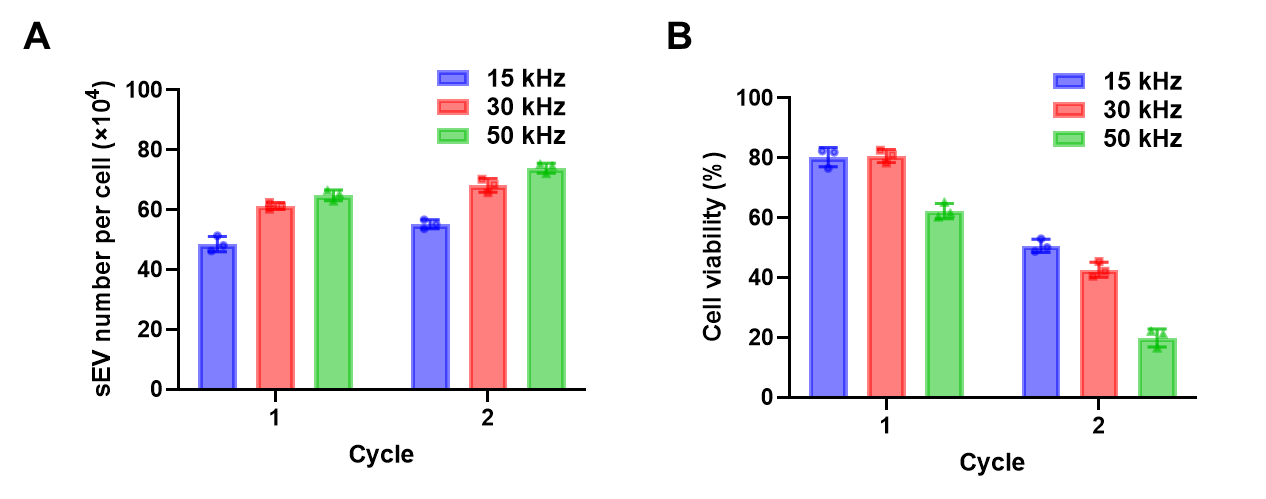


**Figure S1.** The yield of sEVs and viability of MSCs after treatment with different ultrasonic stimulation frequencies and cycles (n=3).


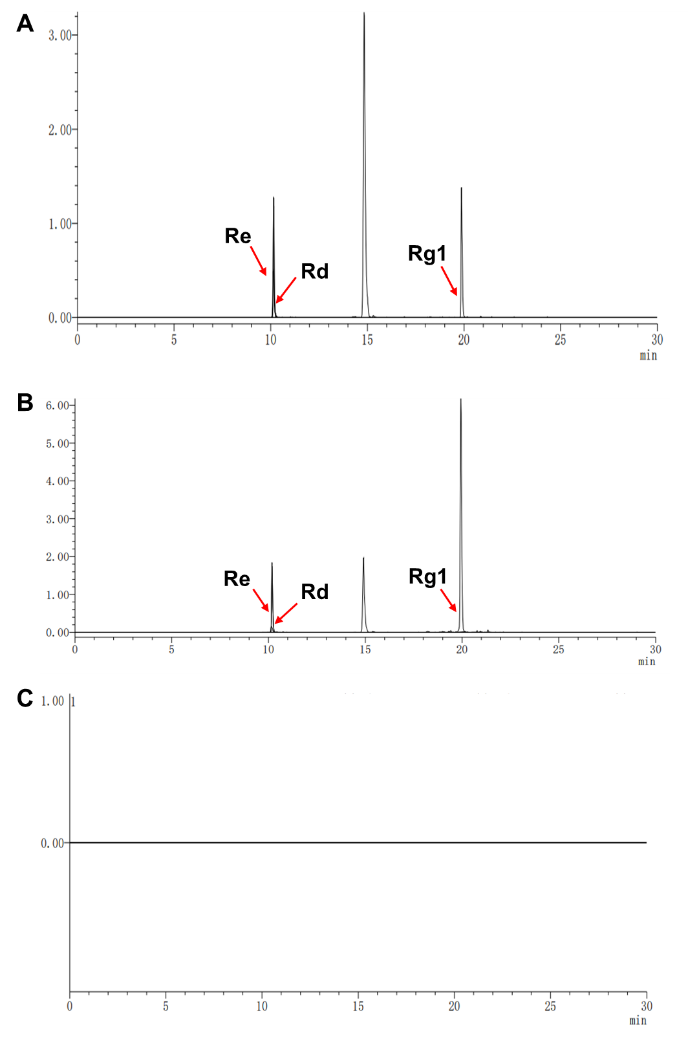


**Figure S2.** Ginsenosides (GS) quantified by Liquid Chromatography-Mass Spectrometry. A) GS Standard. B) GS in GS/sEV. C) Unloaded sEV contents.

**Figure S3.** Viability of HaCaT cells assessed by CCK-8 assay (n=3).

**Figure S4.** Viability of HUVECs assessed by CCK-8 assay (n=3).


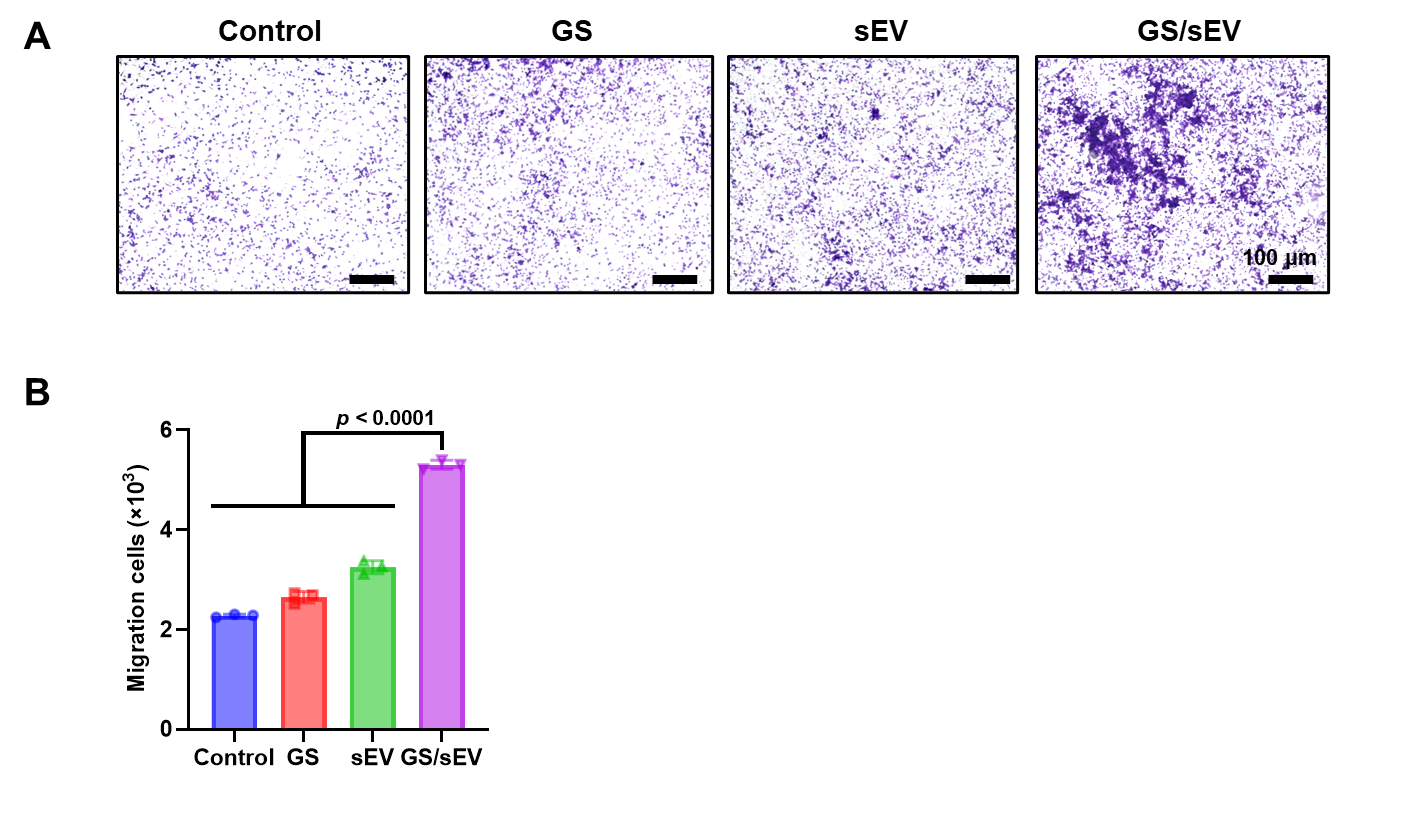


**Figure S5.** Transwell assay showing HUVECs migration at 12 h, 24 h, and 48 h. A) Representative images (scale bar: 200 μm). B) Quantified migration rates (n=3).

**Figure S6.** Quantitative analysis of cell migration rates in the wound healing assay of HaCaT cells (n=3).


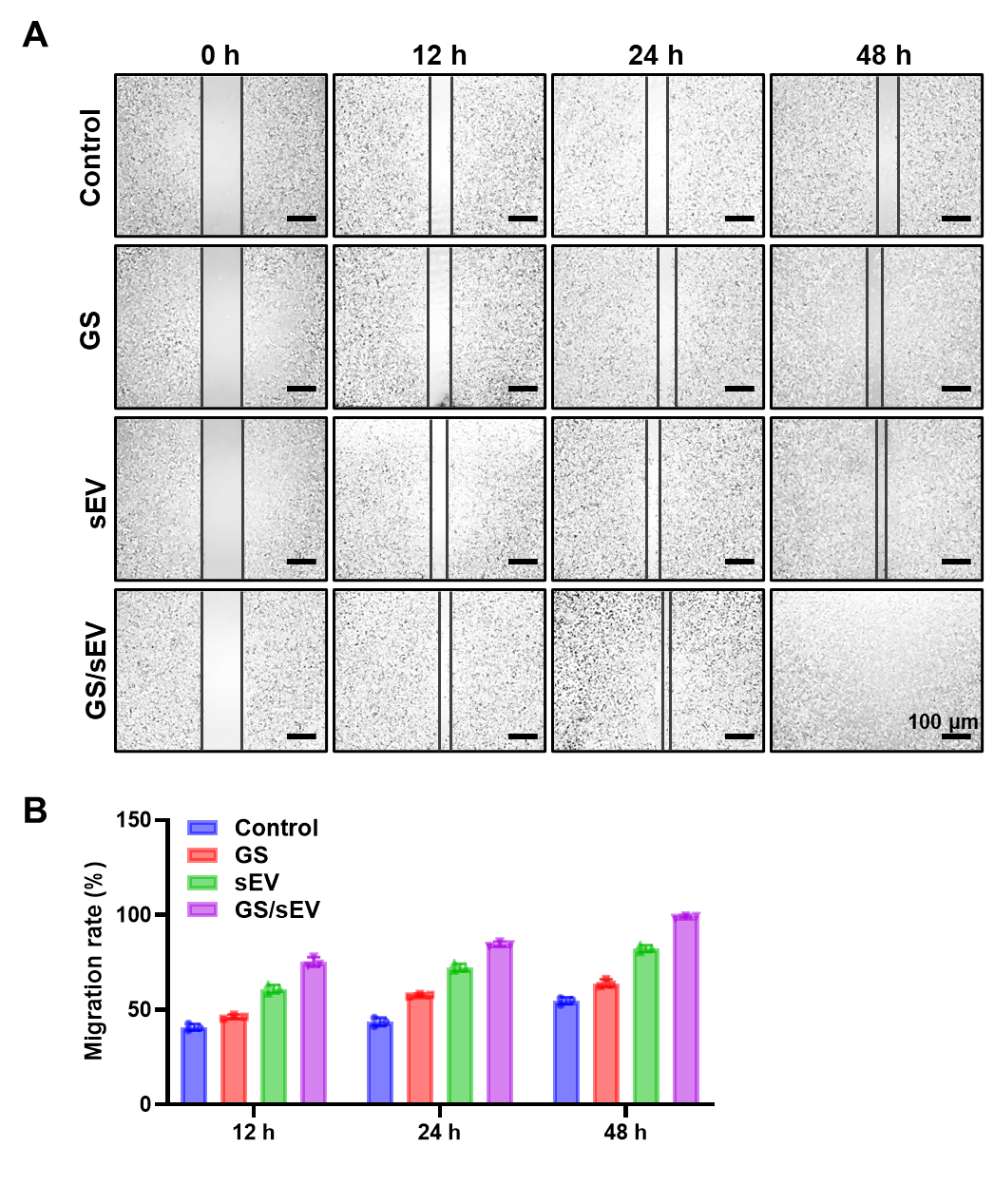


**Figure S7.** Wound healing assay for HUVECs. A) Microscope images at 12 h, 24 h, and 48 h (scale bar: 200 μm). B) Quantitative analysis of cell migration rates (n=3).


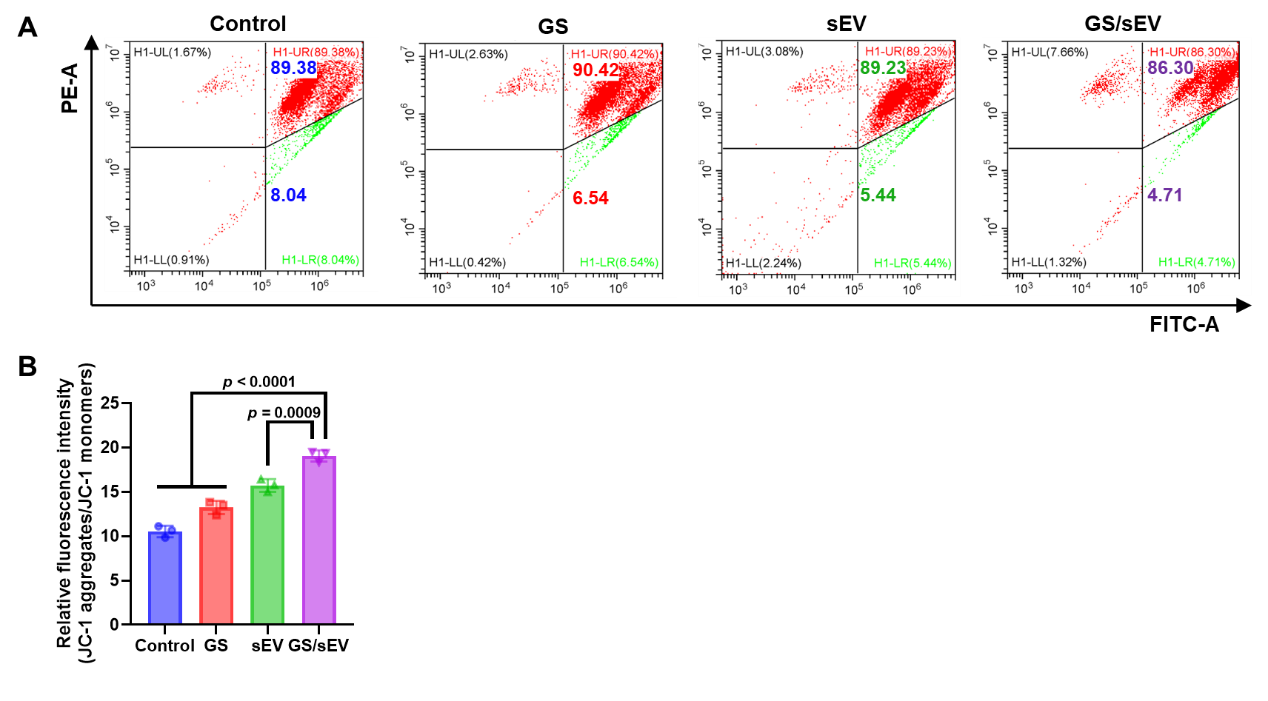


**Figure S8.** Measurement of mitochondrial membrane potential changes in HUVECs analyzed by flow cytometry (n=3).


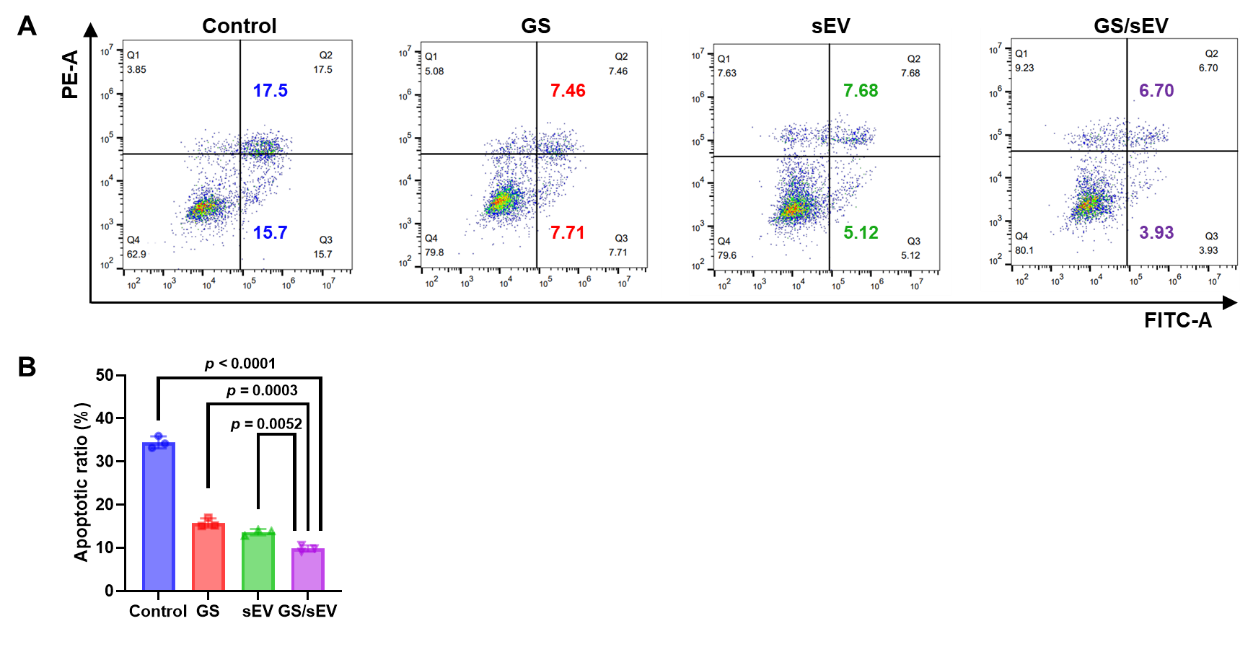


**Figure S9.** Flow cytometry measurement of apoptosis rates of HUVECs in different treatment groups (n=3).


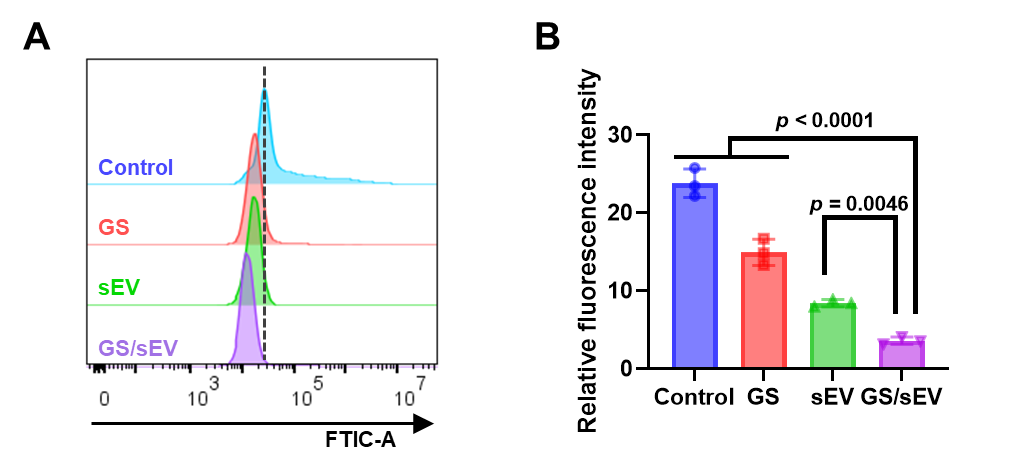


**Figure S10.** Changes in reactive oxygen species (ROS) levels in HaCaT cells analyzed by flow cytometry (n=3).


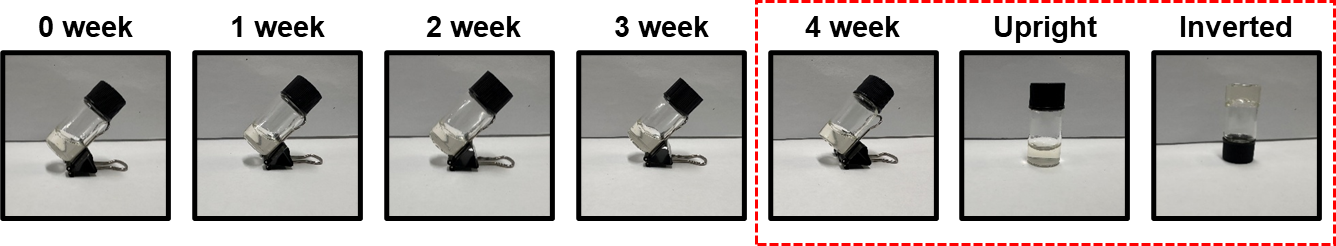


**Figure S11.** Photographs of the morphology of GS/sEV@DNAgel at different time points at -20°C.

**Figure S12.** Evaluation of transdermal permeability for free GS, GS/sEV, and GS/sEV@DNAgel over 48 hours (n=3).

**Figure S13.** Evaluation of skin retention for free GS, GS/sEV, and GS/sEV@DNAgel over 48 hours (n=3).

**Figure S14.** Drug release profiles of free GS, GS/sEV, and GS/sEV@DNAgel under physiological conditions (n=3).


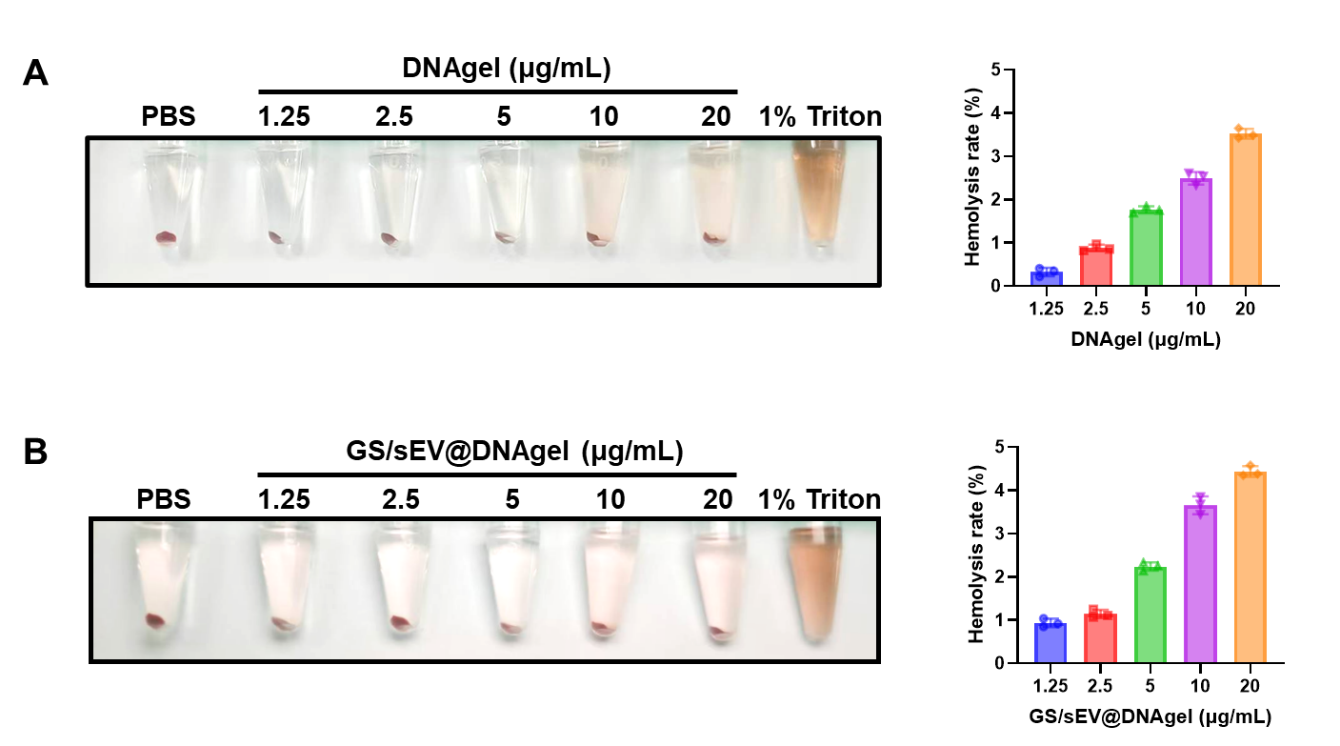


**Figure S15.** Hemolytic assay of hydrogel degradation products (n=3). A) DNAgel. B) GS/sEV@DNAgel.


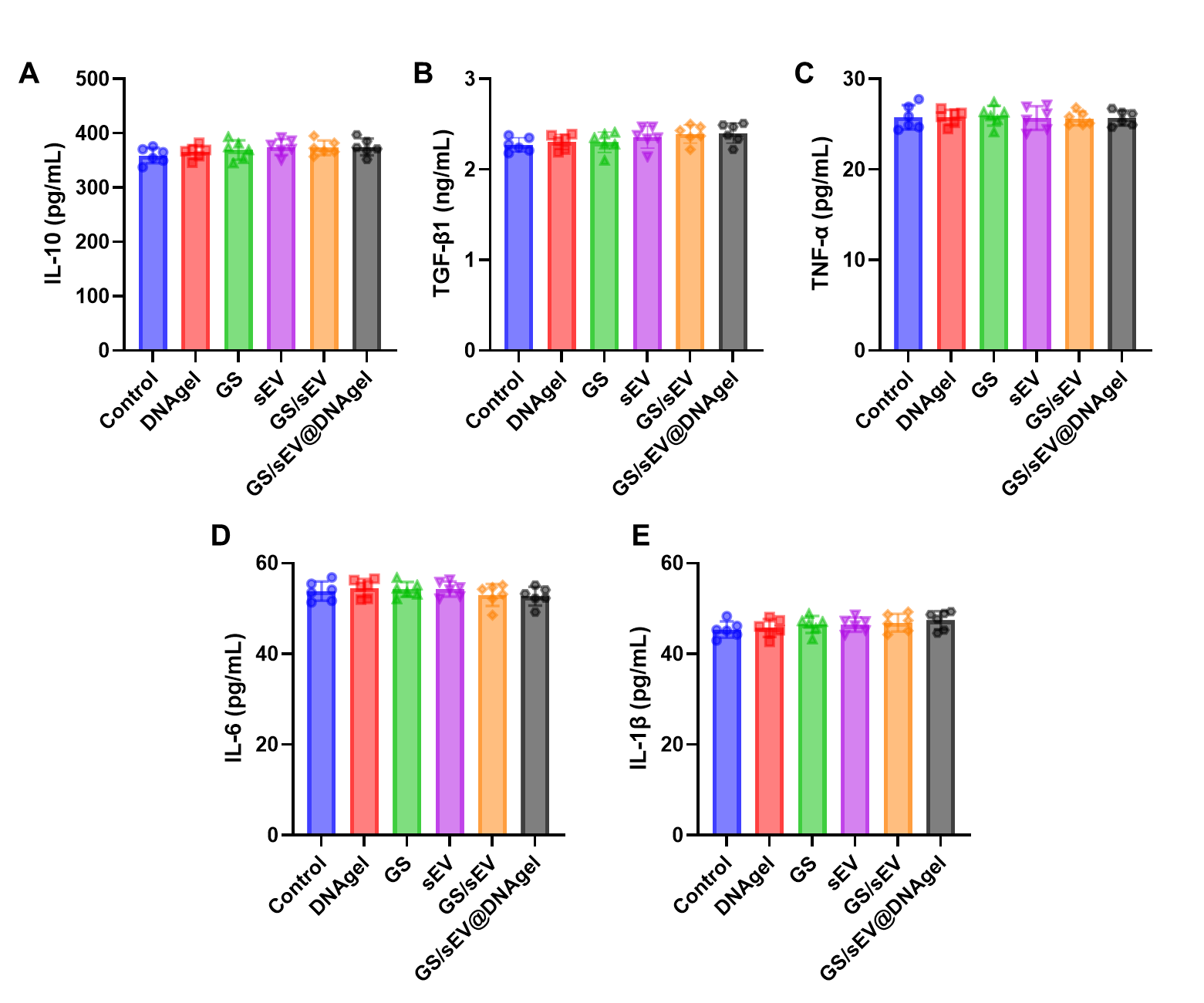


**Figure S16.** Levels of cytokines in serum evaluated by ELISA (n=6). A) IL-10. B) TGF-β1. C) TNF-α. D) IL-6. E) IL-1β.


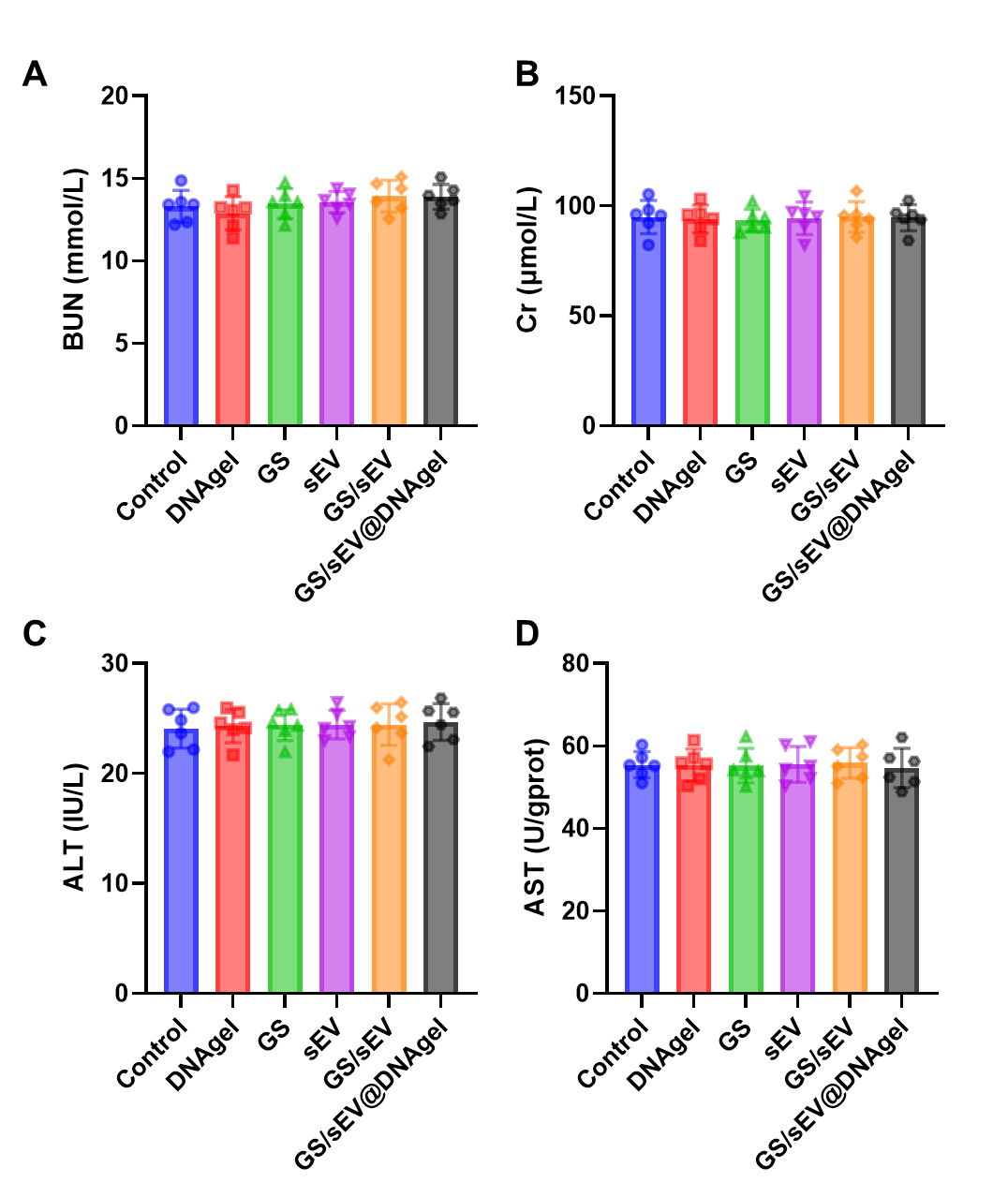


**Figure S17.** Levels of A) BUN, B) Cr, C) ALT, and D) AST in serum (n=6).

**Figure S18.** The relative miR-424 levels in HaCaT cells detected by RT-qPCR (normalized to GAPDH; n=3).


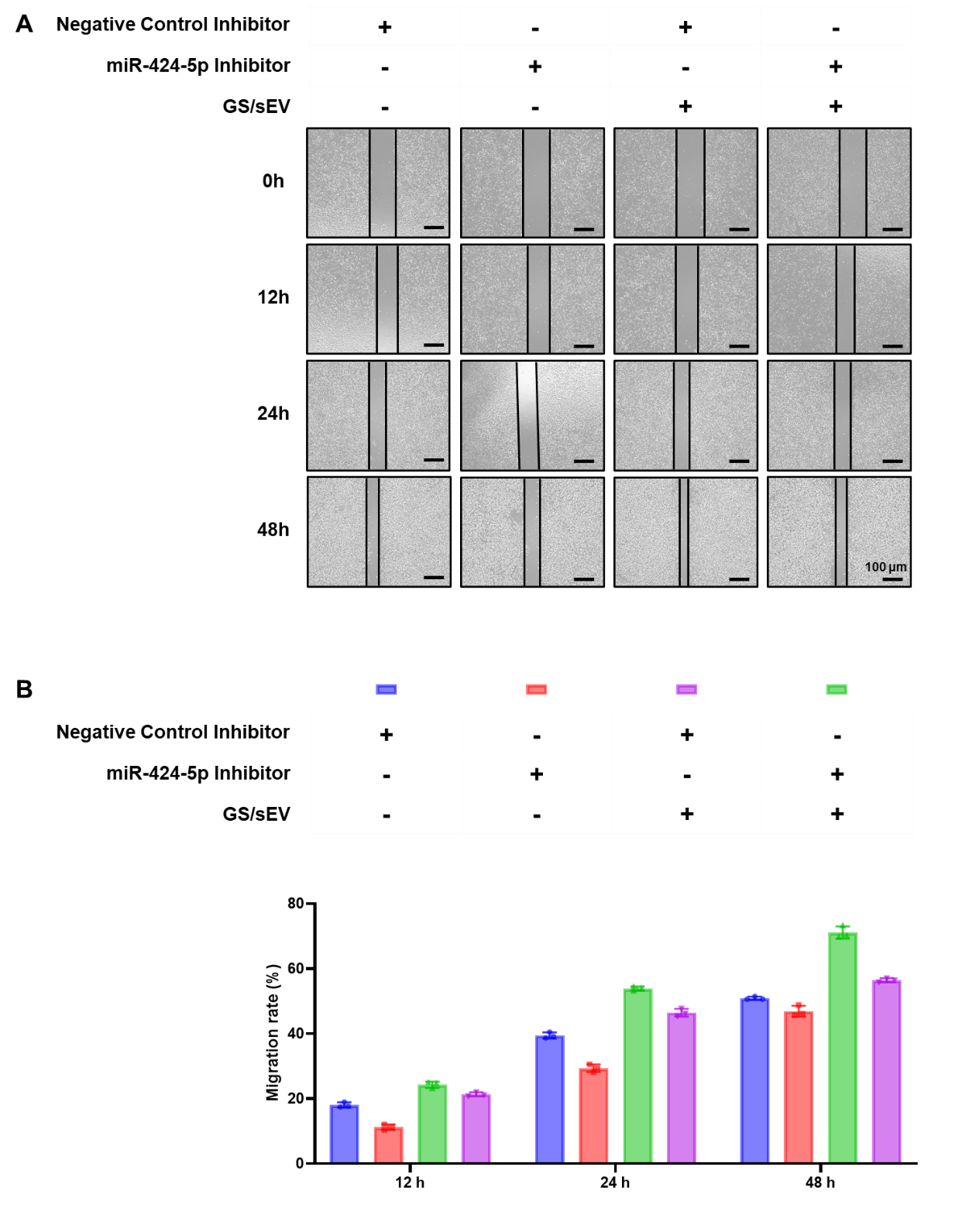


**Figure S19.** Wound healing assay for miR-424-inhibited HUVECs. A) Microscope images at 12 h, 24 h, and 48 h (scale bar: 200 μm). B) Quantitative analysis of cell migration rates (n=3).


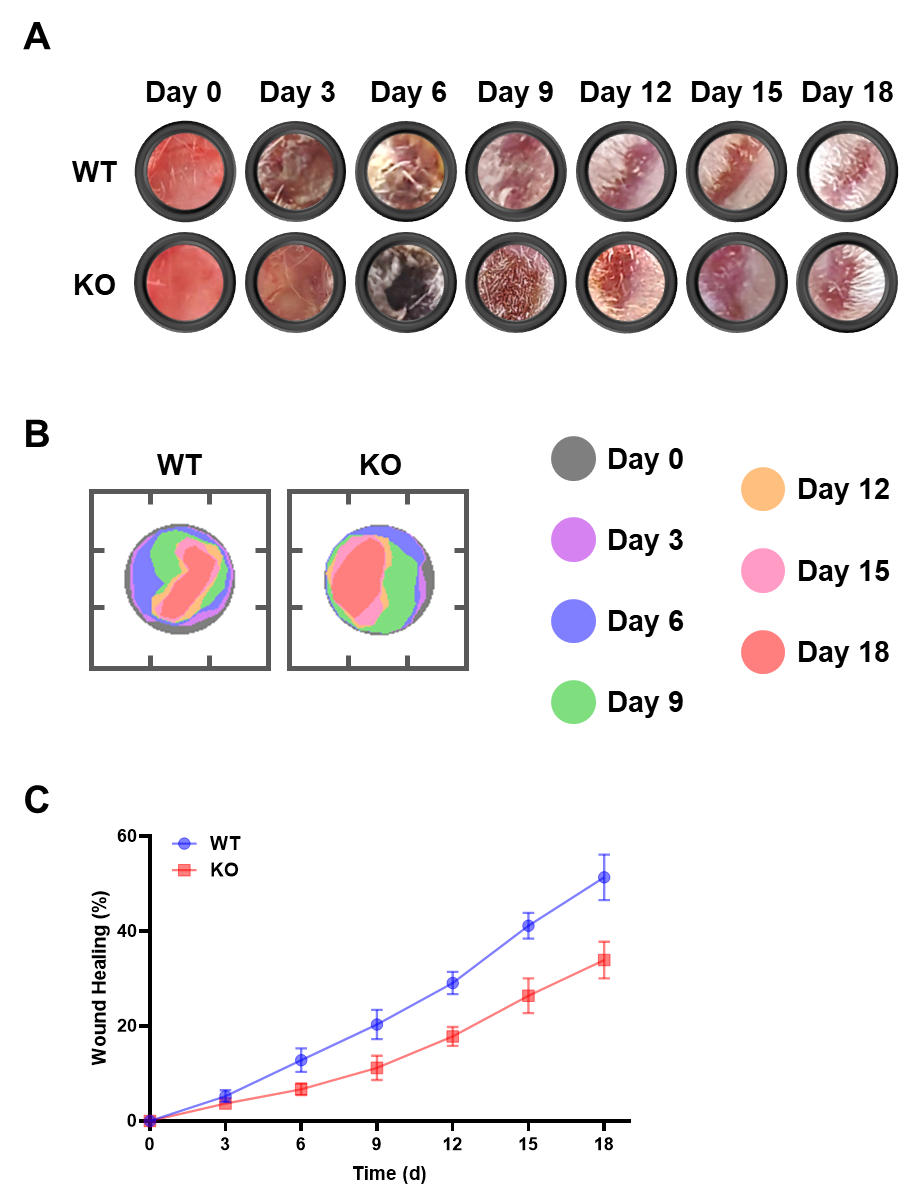


**Figure S20.** MiR-322 knockout delays diabetic wound healing. A) Representative images showing the progression of wound healing at different stages. B) Traces of wound area reduction in different treatment groups. C) Assessment of final wound closure rates (n=6).


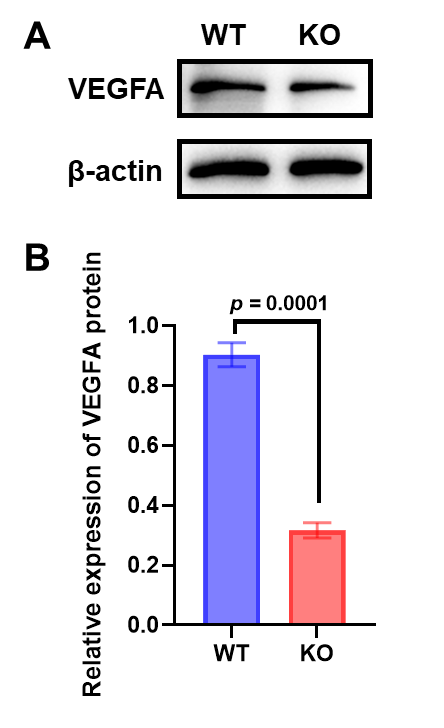


**Figure S21.** VEGFA protein expression in skin tissues of wild-type (WT) and knockout (KO) mice was measured by Western blot assay, band intensities were quantified using ImageJ software, and the expression of VEGFA was normalized to β-actin.

**Figure S22.** The relative VEGFA mRNA levels in skin tissues of wild-type (WT) and knockout (KO) mice were measured by RT-qPCR (normalized to GAPDH; n=6).


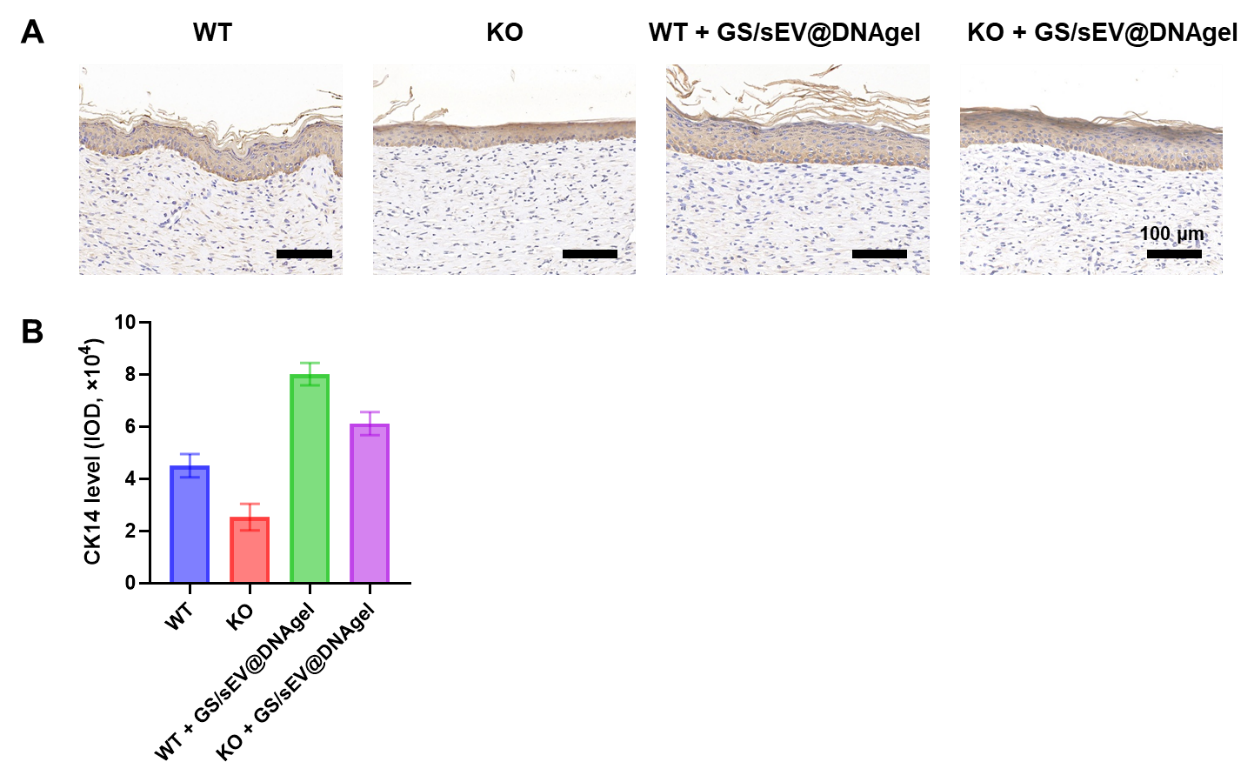


**Figure S23.** A) Immunohistochemical staining for basal keratinocyte marker CK14 protein in skin tissue. B) Quantitative analysis of CK14 expression levels in skin tissue samples from each group.


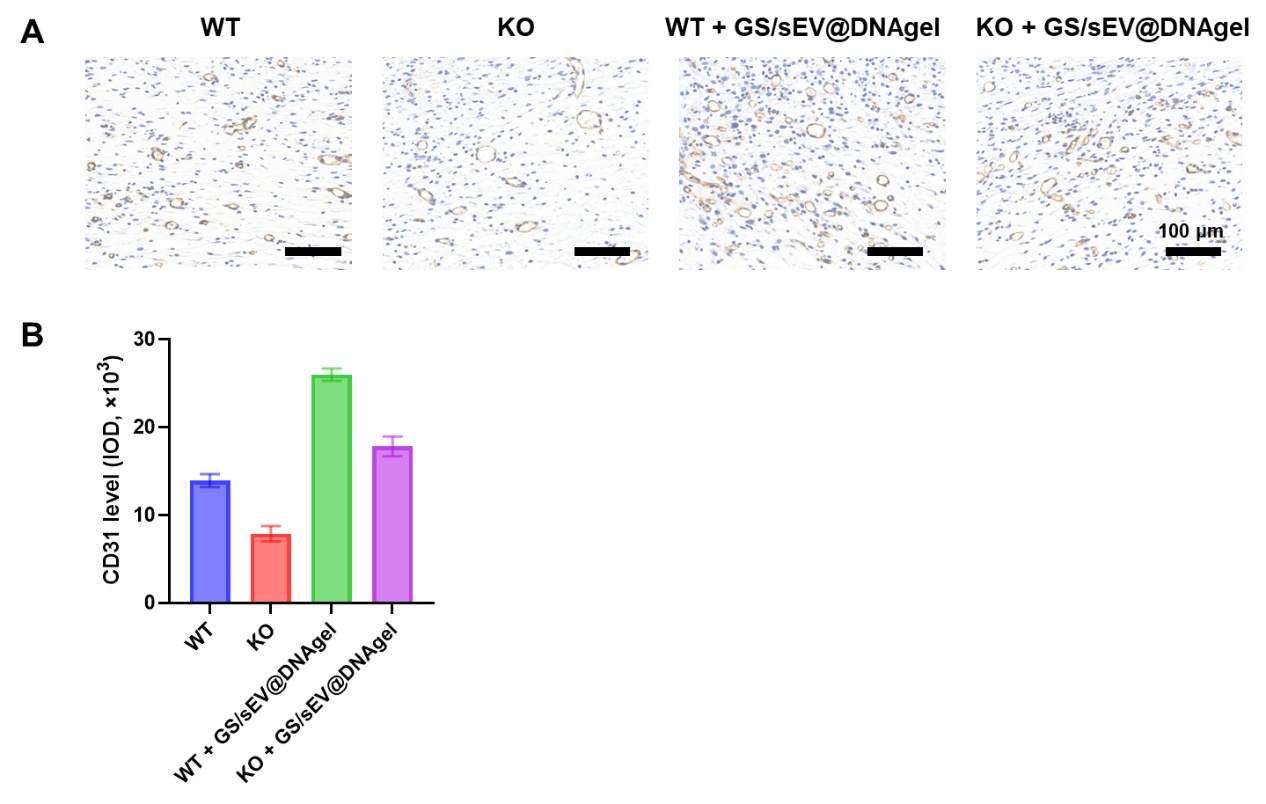


**Figure S24.** A) CD31 expression in skin tissue from each group assessed by immunohistochemistry. B) Quantitative analysis of CD31 expression level.


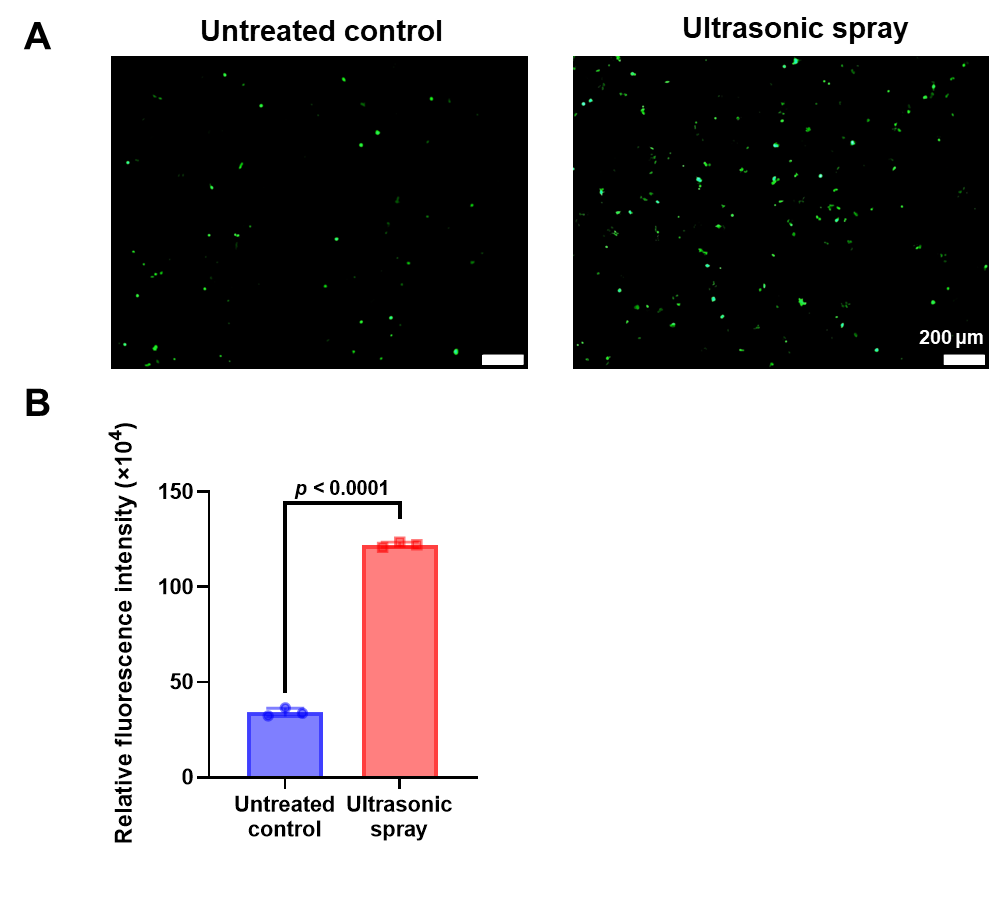


**Figure S25.** Calcium ion concentrations in MSCs detected by the Fluo-4 probe. A) Microscope images (scale bar: 200 μm). B) Analysis of the fluorescence intensity.

**Figure S26.** Membrane fluidity in MSCs (n=3).


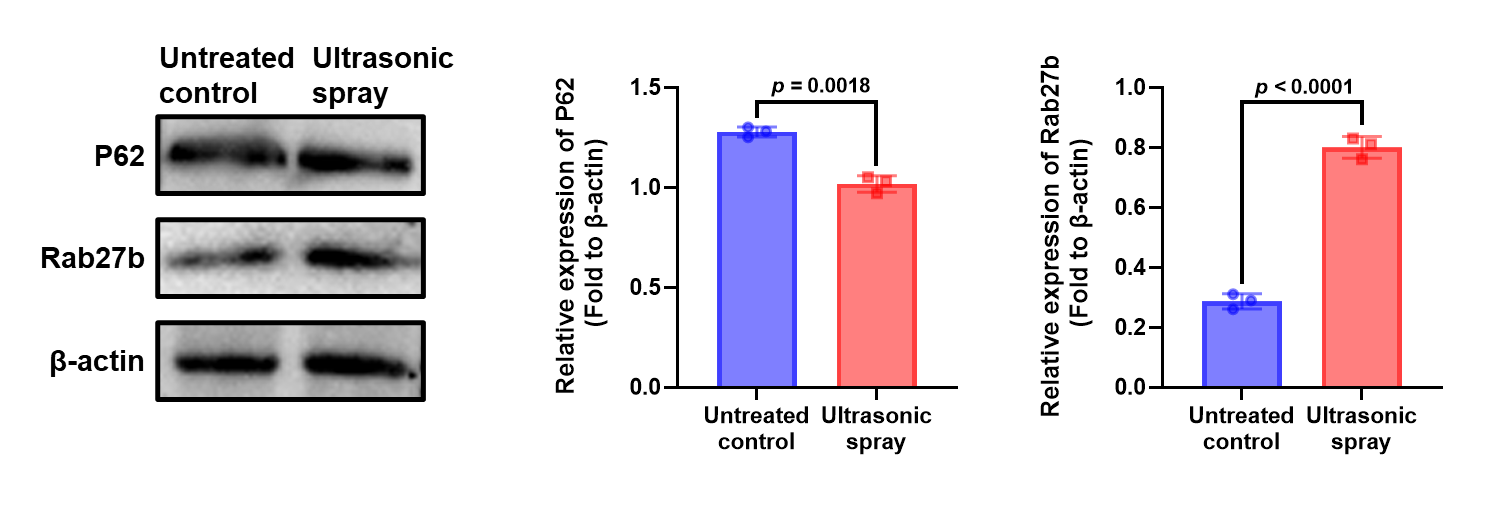


**Figure S27.** Expression of P62 and Rab27b in MSCs, detected by Western blot assay.


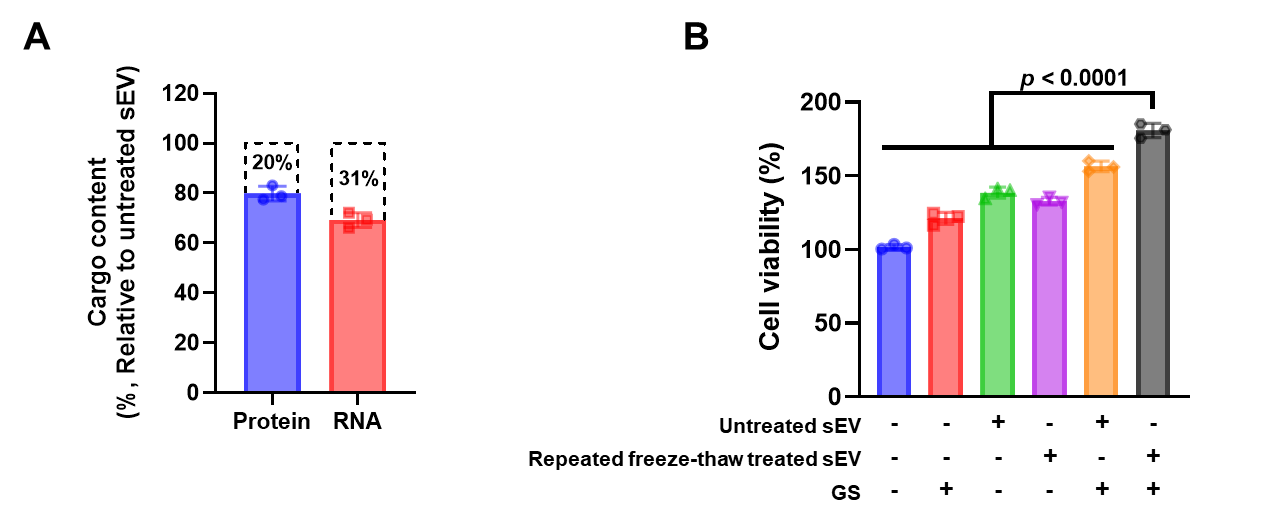


**Figure S28.** Effects of repeated freeze-thaw on sEV. A) Changes in protein and RNA content (n=3). B) Effects of different treatments on HaCaT cells viability (n=3).


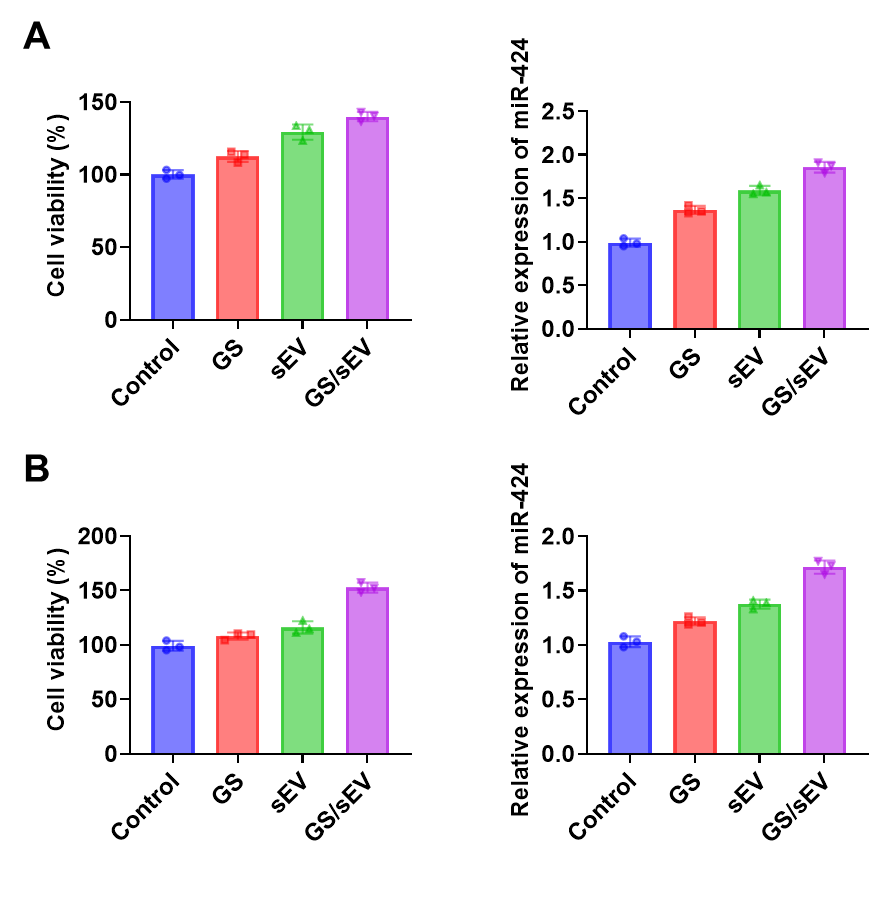


**Figure S29.** Effects of sGS/EV on the cell viability and miR-424 levels in A) human kidney proximal tubular cells (HK-2) and B) human retinal Müller cells.

**T****able S1**. Sequence of nucleotides used for DNA hydrogel synthesis.

| Name | Sequence (5’-3’) |
| --- | --- |
| Y1 | CGATTGACTCTCCACGCTGTCCTAACCATGACCGTCGAAG |
| Y2 | CGATTGACTCTCCTTCGACGGTCATGTACTAGATCAGAGG |
| Y3 | CGATTGACTCTCCCTCTGATCTAGTAGTTAGGACAGCGTG |
| CD63 aptamer-L1 | CACCCCACCTCGCTCCCGTGACACTAATGCTATTTTCATCACGATGGTGAA |
| L1-L2 | GAGAGTCAATCGTTCACCATCGTGATGCATATGAAATCCACT |
| L2 | GAGAGTCAATCGAGTGGATTTCATATG |

**Table S2**. Sequence of primers used in RT-qPCR.

| Name | Sequence (5’-3’) |
| --- | --- |
| VEGFA forward | CGAAGTGGTGGTCATGGATG |
| VEGFA reverse | TTCTGTATCAGTCTTTCCTGGTGAG |
| miR-424 forward | CAGCAGCAATTCATGTTTGAA |
| miR-424 reverse | AACGCTTCACGAATTTGCGT |
| GAPDH forward | AGGTCGGAGTCAACGGATTTG |
| GAPDH reverse | GTGATGGCATGGACTGTGGT |

**Table S3**. Sequence of sgRNAs in miR-322 knockout.

| Name | Sequence (5’-3’) |
| --- | --- |
| sgRNA-A1 | TCGTTGACTCCGAAGGGCTG |
| sgRNA-A2 | CCCTTCGTGGGGAATGTAGA |
| sgRNA-B1 | CTTCGGAGTCAACGAGGGT |
| sgRNA-B2 | ACCTTCTACATTCCCCACGA |
